# Live-Cell Imaging of Vaccinia Virus Recombination

**DOI:** 10.1371/journal.ppat.1005824

**Published:** 2016-08-15

**Authors:** Patrick Paszkowski, Ryan S. Noyce, David H. Evans

**Affiliations:** Department of Medical Microbiology & Immunology and Li Ka Shing Institute of Virology, University of Alberta, Edmonton, Alberta, Canada; University of Utah, UNITED STATES

## Abstract

Recombination between co-infecting poxviruses provides an important mechanism for generating the genetic diversity that underpins evolution. However, poxviruses replicate in membrane-bound cytoplasmic structures known as factories or virosomes. These are enclosed structures that could impede DNA mixing between co-infecting viruses, and mixing would seem to be essential for this process. We hypothesize that virosome fusion events would be a prerequisite for recombination between co-infecting poxviruses, and this requirement could delay or limit viral recombination. We have engineered vaccinia virus (VACV) to express overlapping portions of mCherry fluorescent protein fused to a cro DNA-binding element. In cells also expressing an EGFP-cro fusion protein, this permits live tracking of virus DNA and genetic recombination using confocal microscopy. Our studies show that different types of recombination events exhibit different timing patterns, depending upon the relative locations of the recombining elements. Recombination between partly duplicated sequences is detected soon after post-replicative genes are expressed, as long as the reporter gene sequences are located *in cis* within an infecting genome. The same kinetics are also observed when the recombining elements are divided between VACV and transfected DNA. In contrast, recombination is delayed when the recombining sequences are located on different co-infecting viruses, and mature recombinants aren’t detected until well after late gene expression is well established. The delay supports the hypothesis that factories impede inter-viral recombination, but even after factories merge there remain further constraints limiting virus DNA mixing and recombinant gene assembly. This delay could be related to the continued presence of ER-derived membranes within the fused virosomes, membranes that may once have wrapped individual factories.

## Author Summary

Recombination plays a critical role in DNA repair and also creates the genetic diversity that underpins evolution. This has important implications for viruses, since recombination may create new pathogens with new infectious properties. It has long been known that hybrids can be recovered from cells co-infected with related viruses, some of the first artificial recombinants were produced >50 years ago from variola and rabbitpox viruses. A particular property of poxviruses is that they replicate in membrane-wrapped cytoplasmic structures called “factories”, and each of these factories develops from a single infecting particle. However, if each genome is isolated inside different factories, when and how does the DNA mix to permit recombination? To examine this question, we have developed a fluorescence-based virus recombination assay. Using live cell confocal microscopy, we have timed these reactions and observed that recombinants can be quickly formed when the recombining sequences are located on the same virus genome. However, when the gene fragments are located on different viruses, there is a significant delay (and a reduction) in recombinant gene formation. This delay supports the hypothesis that factories, and the ER-derived cell membranes that surround factories, impede recombination in poxvirus-infected cells.

## Introduction

Genetic recombination serves an essential role as a mechanism for repairing DNA damage, especially the double-stranded breaks that are produced when the replication machinery encounters single-stranded nicks in template DNA. In the field of virology, recombination was first used to define and map bacteriophage genes [1, 2] and is widely used as a tool for genetically engineering a great diversity of viruses. Recombination also affects poxviruses, as was shown by early work with cowpox, variola and vaccinia viruses (VACV) [3, 4]. It was subsequently used to map VACV genes using both classical and marker rescue methods [5–9] and methods developed in the 1980s [10, 11] are also still widely used to produce genetically modified poxviruses.

We, and others, have been studying the mechanism of poxvirus genetic recombination and have observed a process that is capable of generating viruses bearing evidence of multiple genetic exchanges over the course of even a single round of infection [12]. Mechanistically, poxvirus recombination is intrinsically linked to virus DNA replication [13, 14], and VACV recombination is catalyzed, both *in vivo* and *in vitro*, by the viral DNA polymerase (E9) working in conjunction with the I3 single-strand DNA-binding protein [15–17]. These reactions use the polymerase-encoded proofreading 3’-5’ exonuclease activity to initiate an I3-catalyzed single-strand annealing reaction, and the process has been exploited for its commercial utility as an *in vitro* tool for cloning DNA [18]. Recombination has great biological relevance as it generates the genetic variation that is the substrate for viral evolution. For example, traditional smallpox vaccines comprise a genetically diverse quasispecies, wherein every virus exhibits evidence of having undergone inter- and intra-molecular recombination during its evolution [19]. Analysis of variola genome sequences suggests that recombination may also have shaped the evolution of this pathogen [20]. More recently it has been speculated that an “accordion-like” gene duplication and reduplication process [21, 22] could also promote the evolution of potentially essential poxvirus genes. Although clearly illustrating a variant form of virus DNA recombination-repair, the mechanistic details remain poorly understood.

One of the characteristic features of poxvirus biology is that as virions enter the cell, each infecting particle initiates the formation of separate replication sites or “factories” [23, 24]. We have shown that these structures mix inefficiently [25], which may explain the seemingly contradictory observation that although hybrid viruses are not produced in great abundance in cells co-infected with different viruses, the recombinants that are formed appear to have undergone a lot of recombination [12]. Presumably, each replication site has the potential to catalyze efficient recombination, but if the DNA in different virus factories doesn’t mix, then there is no opportunity to produce recombinant virus progeny. Poxvirus factories are thought to be bounded by membranes (most likely) derived from the endoplasmic reticulum (ER) [26], and the DNA in different factories doesn’t seem to mix until relatively late in the infectious cycle [25].

That said, these statements incorporate some assumptions that have yet to be proven. One feature of these processes that has not been clearly established is how the timing of recombination relates to visible features of the virus life cycle. We presume that factory fusion reactions would have to precede recombinant virus production, but this has not been formally demonstrated beyond correlation analysis. We are also presuming that intracellular dynamics and mixing efficiency, rather than enzymology, is what constrains recombinant virus production. The necessary enzymes (E9 and I3) would be present from the start of factory development, but we cannot exclude the possibility that the catalytic capacity to produce mature recombinants isn’t fully active until late in infection. If that were the case, the timing of recombinant production would not be dependent solely upon geometrical constraints.

To explore these questions, we have employed a technology used previously to track factory development [25]. Cells were constructed that constitutively expressed the bacteriophage lambda cro protein fused to enhanced green fluorescent protein (EGFP-cro). Upon infection with VACV, the EGFP-cro protein migrates from the nucleus and labels the virus DNA in the growing factories. This permits live cell imaging of virus development over the course of infection. In this study we have incorporated genes encoding cro fused to monomeric cherry fluorescent protein (mCherry-cro) into VACV. By apportioning overlapping fragments of the mCherry-cro gene into different viruses, and co-infecting EGFP-cro cells with these viruses, we can track both factories and recombinant production using green and red fluorescence, respectively.

Our studies show that different types of poxvirus recombination events exhibit different timing patterns, depending upon the relative locations of the recombining elements. Recombination between partly duplicated sequences is detected soon after post-replicative genes are expressed, as long as the reporter gene sequences are located *in cis* within an infecting genome. The same kinetics are also observed when the recombining elements are divided between a virus and transfected DNA. In contrast, recombination is significantly delayed when the recombining sequences are located *in trans*, on different co-infecting viruses, and mature recombinants aren’t detected until well after late gene expression is well established. The delay is consistent with the hypothesis that virus factories create one impediment to inter-viral recombination, but even after factories merge there remain further constraints limiting recombinant production.

## Results

### Cell lines and reporter viruses

We have previously shown that a molecule composed of enhanced green fluorescent protein fused to the phage lambda cro DNA-binding domain (EGFP-cro) provides a useful tool for tracking DNA *in vivo*. When the EGFP-cro protein is expressed constitutively from a cellular promoter, it diffuses freely to sites of VACV DNA replication and permits tracking of viral “factories” [25]. In this study we have used a modification of this approach, to examine when and where recombinant poxviruses are formed during poxvirus infection, and thus permit optical tracking of virus recombination in real time.

**Fig 1:**
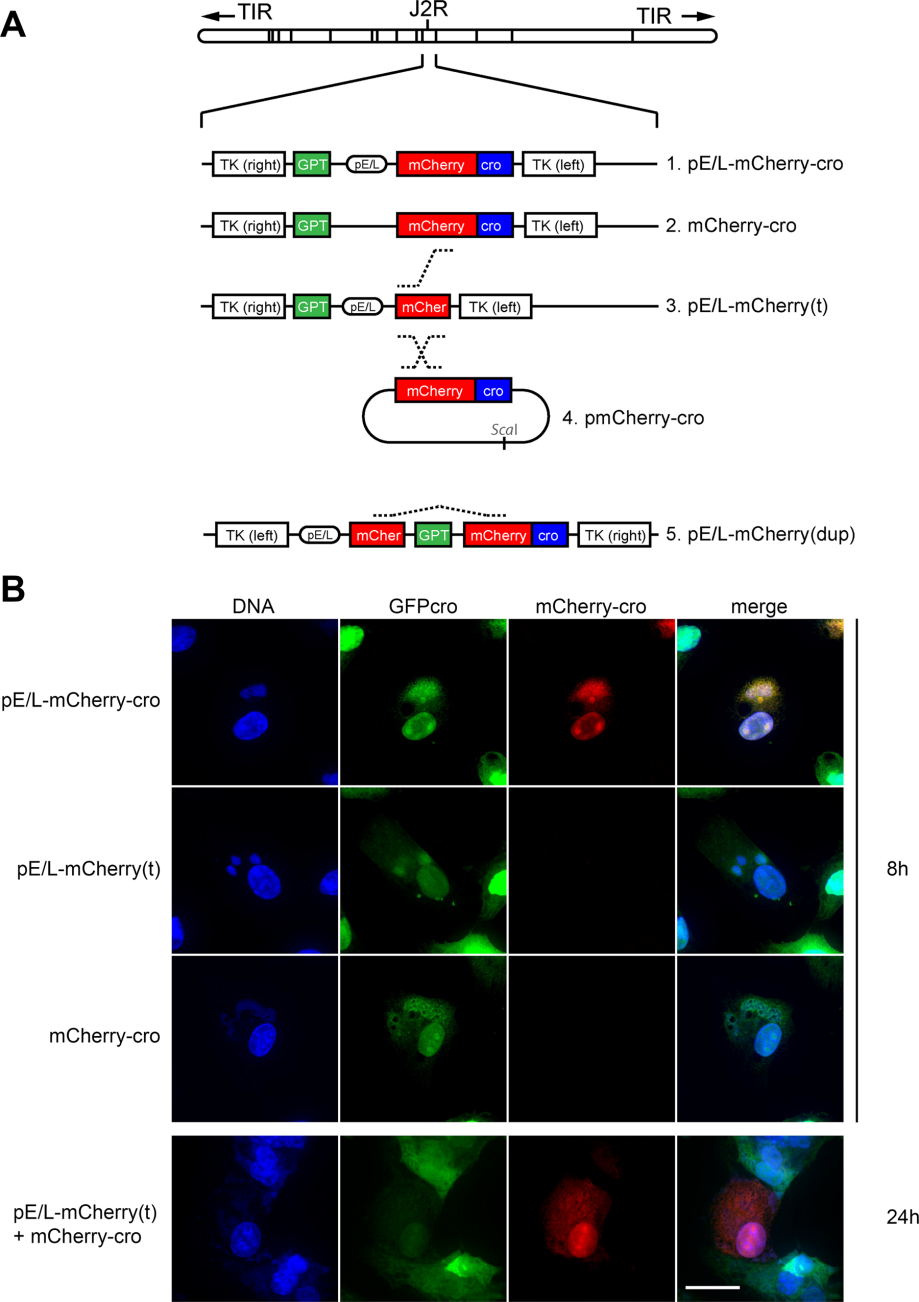
Characterization of the recombinant VACV constructed for this study. (A) Four recombinant viruses were constructed encoding combinations of the mCherry and Cro genes with or without a synthetic early-late pox promoter (pE/L), all inserted into the TK locus of WR VACV. For convenience, these are shown in the conventional orientation, but the inserts [with the exception of the pE/L-mCherry(dup) virus] are actually inverted relative to the virus genome. Recombinants were selected using mycophenolic acid resistance and/or mCherry fluorescence. (B) Subcellular localization of fluorescent labels relative to viral factories. EGFPcro BSC-40 cells were infected at an MOI = 5 with the indicated viruses. At the indicated times, the cells were fixed and stained for total viral and cellular DNA using DAPI. Images were collected using a spinning disc confocal microscope at 60× magnification. The scale bar = 15 μm.

The principles behind these assays, and the viruses used in the different studies are shown in Fig 1. These viruses encode all (or parts) of a gene comprising a poxvirus early-late promoter (pE/L) driving expression of mCherry fluorescent protein fused to a cro DNA-binding peptide (mCherry-cro). The hybrid promoter combines conserved sequence elements that have been traditionally defined as driving either immediate early or late gene expression. It is not expected to be active at intervening time points. All of the gene constructs were incorporated into the non-essential VACV thymidine kinase locus. The mCherry protein was chosen for these studies because it is bright, and folds rapidly after being transcribed and translated [t_1/2_ = 15 min (Clontech)]. We incorporated the cro DNA-binding domain to concentrate the signal and in the hope that the fusion protein might, at least transiently, selectively target the virus factory from where it had originated. Subsequent studies showed that the protein does concentrate on DNA, but also still diffuses freely throughout infected cells, as judged by red fluorescence in the infected cell nucleus.

The virus designated as pE/L-mCherry-cro served as a control for reference purposes (Fig 1A). When BSC-40 EGFP-cro cells were infected with this virus for 8 h, we detected a strong mCherry signal, co-located with DAPI and EGFP-cro at sites of virus replication and within the nucleus (Fig 1B, top). At this time point VACV factories were typically starting to expand in volume and the initial punctate appearance was beginning to blur as the virus transitioned into the later stages of the infection cycle. Virus #2 (mCherry-cro) encodes an intact mCherry-cro fusion protein, but lacks the E/L promoter, while virus #3 (pE/L-mCherry[t]) encodes the E/L promoter driving a truncated and non-fluorescent mCherry protein. In contrast to cells infected with the control virus, no mCherry signal was detected in cells separately infected with viruses #2 and #3 even though they were clearly infected judging by the recruitment of EGFP-cro protein to DAPI-stained virus factories (Fig 1B, middle rows; VACV-mCherry-cro [S1 Video; VACV pE/L-mCherry(t) [S2 Video]). To test whether this system could detect recombinant virus production, the BSC-40 EGFP-cro cells were co-infected with a 1:1 mixture of the pE/L-mCherry(t) and mCherry-cro viruses, at a total MOI = 5 (i.e. MOI = 2.5 for each virus). Little red fluorescence was seen at the 8 h time point, but by 24 h red fluorescence was detected in many of the cells (Fig 1B, bottom panel). These data showed that this method can be used to detect VACV recombinants, but the process is a slow one and recombinant gene products aren’t detected until late in the infection cycle. This matter is examined in greater detail in the sections to follow.

We also examined how well these viruses would grow, using single-step growth curves. All of the viruses grew initially at nearly the same rate in BSC-40 cells, although the pE/L-mCherry-cro control yielded 10-to-60-fold less progeny than the other viruses (S1 Fig). The mCherry-cro protein is produced in abundance by the pE/L-mCherry-cro virus, and appears to be packaged into virions. Because of this, it probably has a somewhat deleterious effect on virus growth (or packaging) over multiple rounds of VACV replication.

### Recombination between two co-infecting VACV

We used live cell imaging to track the growth and movement of separate viral factories, in order to compare these events with the time(s) when recombinant mCherry can first be detected. In designing these experiments, we were cognizant of the fact that the timing of these events depends upon the sensitivity of the experiment (i.e. the time when one can first detect fluorescence), and thus the strength of the mCherry signal. Therefore we set the gain in all of these experiments, at a level that would detect the weaker late mCherry signal observed in cells co-infected with pE/L-mCherry(t) and mCherry-cro viruses. In order to standardize the timing between different experiments, we defined the “factory time” t_f_ = 0:00 as being the time (in hours) when small punctate cytoplasmic viral factories were first detected by EGFP-cro staining. We presume that these would be uncoated particles, since the DNA is accessible to cytoplasmic EGFP-cro protein, and they were detected 1–3 h post-infection. We defined T_i_ as the time (in hours) after the addition of virus.

**Fig 2:**
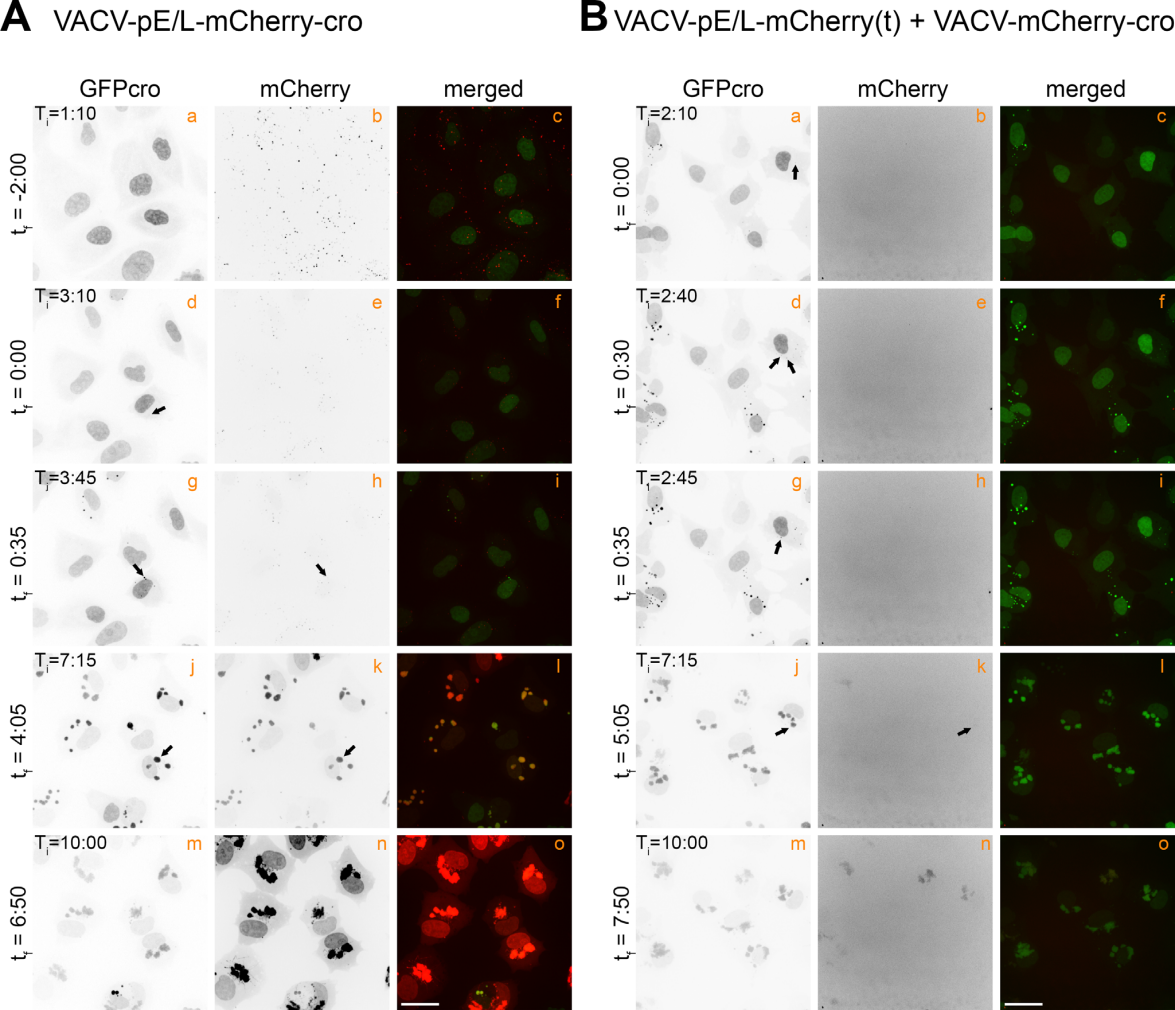
Tracking the appearance of virus-encoded mCherry proteins. (A) EGFPcro BSC-40 cells were infected at a MOI = 5 with VACV-pE/L-mCherry-cro, and the red and green fluorescence then tracked over time, collecting images 5 minutes apart, across 10 different fields (only a single representative field is shown). These are stills, the complete time-lapse movie is found in S3 Video. (B) EGFPcro BSC-40 cells were co-infected at a total MOI = 5 with VACV-pE/L-mCherry(t) and VACV-mCherry-cro viruses, and tracked using live cell microscopy to detect the appearance of recombinant mCherry-cro protein. The panels show different stills taken from S4 Video. Note the delay in the appearance of a mCherry-cro signal compared to panel (A). The scale bar = 25 μm.

VACV pE/L-mCherry-cro was used as a control to establish when an intact mCherry reporter protein is first expressed during virus infection. This was complicated by the fact that many punctate mCherry^+^ signals were detected at the earliest time points, prior to entry and uncoating, and long before the first appearance of any EGFP-cro labeled factories (Fig 2A, t_f_ = -2:00). This mCherry signal was only seen transiently and probably comprised mCherry-cro protein that had been incorporated into the virus particles used in the inoculum. It was mostly lost by degradation and/or dilution as the virus entered the cell and the DNA uncoated (S3 Video). The first intracellular EGFP-cro-labeled virus particles were detected ~3 h post-infection (Fig 2A, panel d) and these acquired a secondary mCherry fluorescent signal only a few minutes after first detecting the viral factories (Fig 2A, t_f_ = 0:35, panel h). As the infection progressed the intensity of the EGFP and mCherry signals increased, indicative of active replication and new mCherry synthesis. The factories also moved around and started to coalesce into larger assemblies by T_i_ = 7:15 (Fig 2A, panels j and k). Because the gain was set to detect the faint signals produced by other combinations of virus, as noted above, the mCherry signal started to saturate the detectors in the later parts of the experiment (Fig 2A, T_i_ = 10:00, panels n and o).

Quite different mCherry expression kinetics were seen in cells co-infected with the pE/L-mCherry(t) and mCherry-cro viruses. The cells were infected with the two viruses at a combined MOI = 5, and imaged to again track the development of EGFP- and mCherry-tagged viral factories (Fig 2B; S4 Video). No detectable mCherry signal was seen either in the inoculum or within a few minutes of first detecting the EGFP-labeled factories (Fig 2B, panels b, e, and h). As in cells infected with the control virus, these factories gradually migrated towards the nuclear periphery and started to merge into a shared structure, around t_f_ = 0:35 in the example shown here (Fig 2B, compare panel d to panel g). To confirm that the viral factories had indeed fused, we quantified the fluorescence intensities of individual factories before and after fusion (S2 Fig). This method [25] showed that factory fusion was associated with the conservation of the sum of the fluorescence intensities exhibited by the two separate factories prior to fusion, plus a correction for the replication and EGFP-cro accumulation over the 5-minute interval between frames. However, although one sees abundant evidence of factories fusing, a mCherry signal was still not detected until a larger aggregate had formed by the t_f_ = 5:05 time point (Fig 2B, panel k). Thereafter, this mCherry signal gradually gained intensity and was distributed across all EGFP-tagged cytoplasmic viral factories. These particular viruses, and this approach, illustrate two features of VACV recombination *in vitro*. First, recombinant genes aren’t detected until long after the different factories have started to fuse and mix their DNA. Secondly, even after factory fusion takes place, there is a further delay before a recombinant signal is detected.

### Timing of recombinant signals

**Fig 3:**
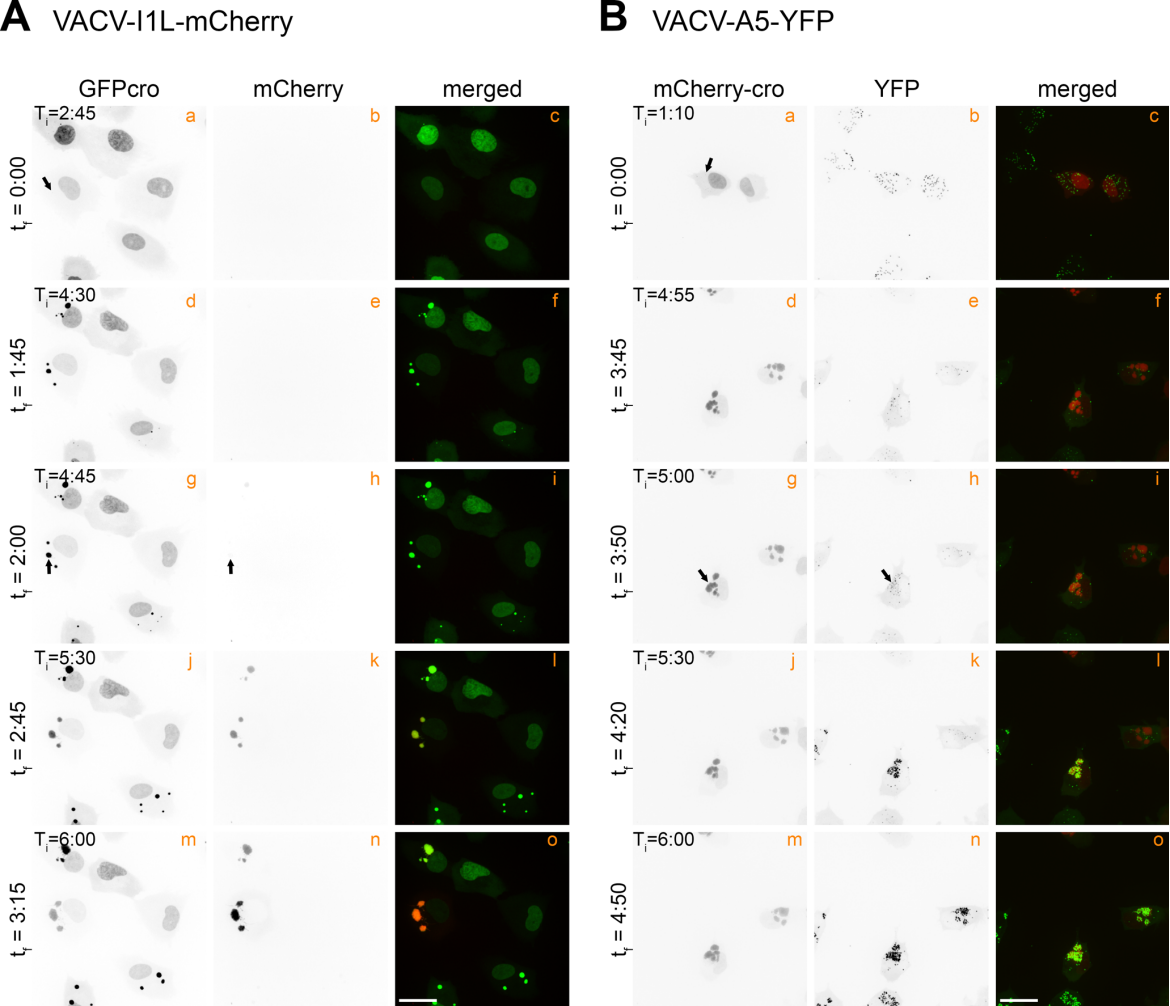
Timing of expression of VACV post-replicative and late genes. (A) EGFPcro BSC-40 cells were infected at a MOI = 5 with a virus encoding mCherry-tagged to the I1 protein (VACV-I1L-mCherry) and then tracked via live cell microscopy. These are stills taken from S5 Video. (B) mCherry-cro BSC-40 cells were infected and imaged as in (A) except using a YFP-tagged A5 virus (VACV-A5-YFP). These are stills taken from S6 Video. The I1L and A5L genes are post replicative and late genes, respectively. The scale bar = 25 μm.

Several additional viruses were used to determine how the timing of recombinant virus detection relates to other stages in VACV development. I1L is representative of a class of VACV genes called post-replicative genes [27]. We assembled a VACV encoding I1 protein, under its native promoter, and fused to a mCherry reporter protein. This recombinant protein was first detected at t_f_ = 2:00 (Fig 3A, panel h; S5 Video), and as expected, that is later than the “early” fluorescent signal detected in cells infected with the pE/L-mCherry-cro virus (Fig 2A, panel h; t_f_ = 0:35). We also measured the timing of expression of an A5L-YFP fusion protein. A5L is regulated by a late viral promoter and newly expressed YFP was not detected until t_f_ = 3:50 (Fig 3B, panel h; S6 Video). This time point still significantly precedes the timing of the appearance of a mCherry signal (t_f_ = 5:05) in cells co-infected with the pE/L-mCherry(t) and mCherry-cro viruses.

**Fig 4:**
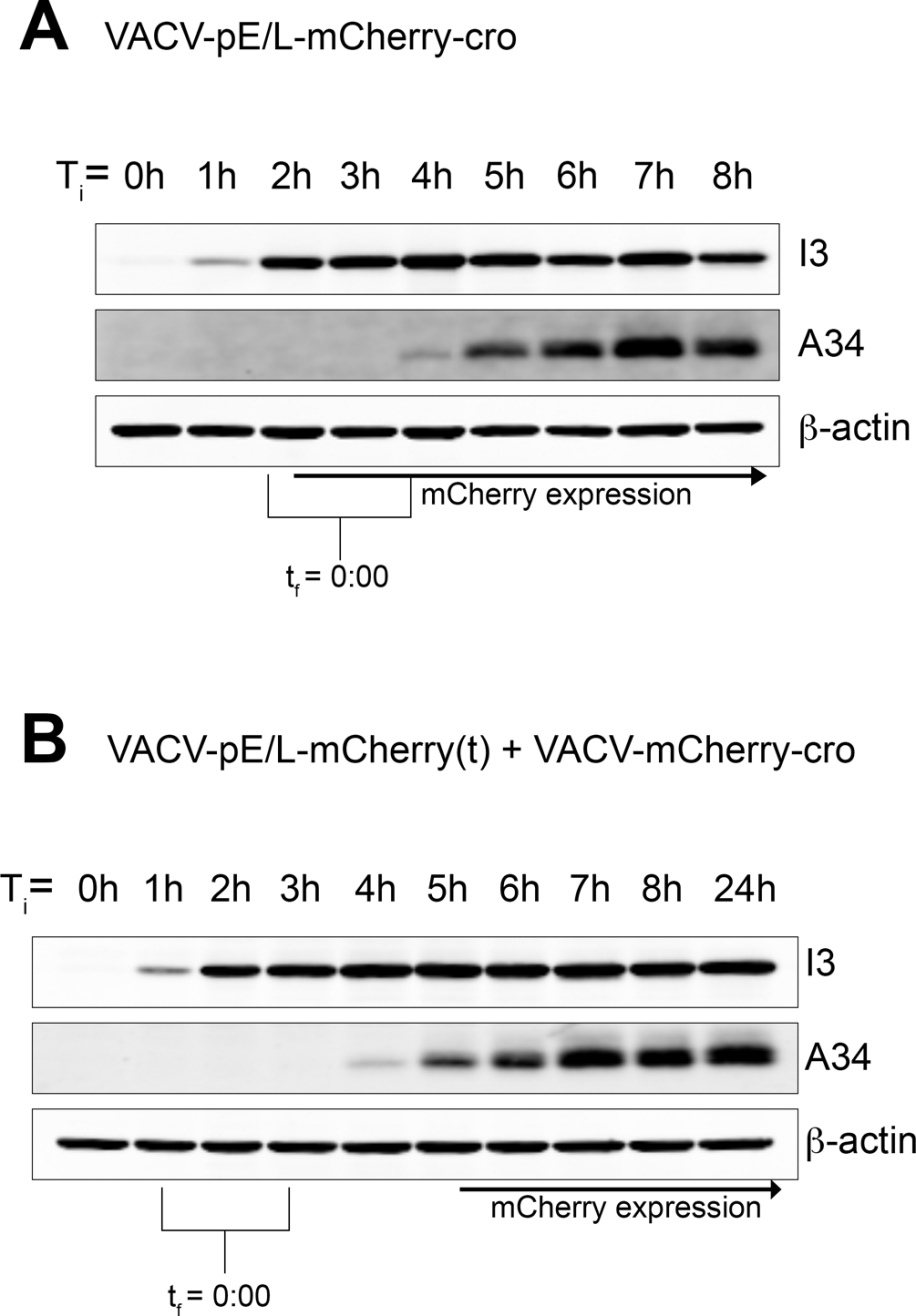
Timing the appearance of early (I3) and late (A34) genes by western blotting. EGFPcro BSC-40 cells were cultured in 60 mm dishes, infected with the indicated viruses [(A) VACV-pE/L-mCherry-cro; (B) VACV-pE/L-mCherry(t) + VACV-mCherry-cro], and then different dishes were harvested at the indicated times. Protein extracts were then prepared and western blotted for the indicated proteins. In parallel, the same viruses were used to infect EGFPcro BSC-40 cells on Fluorodish slides, transferred to a confocal microscope, and imaged over time. T_i_ is the time of initiating infection (in both arms of the experiment) and t_f_ is the time from factory formation, determined microscopically. Although I3 and A34 appear with essentially identical early and late kinetics, respectively, in both infections, the mCherry signal is greatly delayed in cells infected with VACV-pE/L-mCherry(t) + VACV-mCherry-cro viruses and appears only after A34 expression is first detected.

To confirm the timing of early and late gene expression by independent methods we also used ordinary Western blotting. Cells were infected with the pE/L-mCherry-cro virus or co-infected with the pE/L-mCherry(t) plus mCherry-cro viruses. Samples were collected every hour and probed to detect another highly expressed early gene product (I3) and a late one (A34). The timing of mCherry expression was determined by microscopy, because the low levels of mCherry that are easily detected optically (for timing purposes) aren’t as easily detected by Western blotting. Although the timing determined by these methods is a bit less accurate, I3 was first detected at approximately T_i_ = 1 h and A34 at about the T_i_ = 4–5 h mark. When we normalize the data to a common “start” point by marking the time where factories first form (t_f_ = 0:00) in each microscopy experiment and aligning it with the time of initiating infection (T_i_ = 0:00), it is again evident that mCherry is expressed early during pE/L-mCherry-cro virus infection (as expected), whereas it is expressed late or very late in co-infected cells (Fig 4).

### Viral factory fusion significantly delays the time to generate VACV recombinant viruses

The results outlined above are perhaps not surprising as the promoter in the pE/L-mCherry-cro virus permits mCherry expression prior to uncoating (early). In contrast any recombinants that are assembled after that point can’t be detected until late gene expression is initiated. Does the very late appearance of a mCherry signal in co-infected cells simply reflect constraints imposed by transcriptional patterns, or is this truly due to recombinants being assembled and matured very late in infection? We used two approaches to investigate this question.

In the first approach we took the two overlapping fragments of the pE/L-mCherry-cro gene that are encoded separately on the pE/L-mCherry(t) and mCherry-cro viruses, and incorporated them into a single virus separated by a drug-selectable marker (Fig 1A, pE/L-mCherry(dup); S7 Video). Although the tandem duplication is unstable, mixed stocks of parental and recombinant viruses can be obtained by continued selection for the drug-resistance marker. These two kinds of viruses can be differentiated, based on the fact that any pre-existing recombinants in the virus stocks should begin to synthesize mCherry-cro protein immediately after uncoating, while the presence of parental (i.e. non-recombined) VACV pE/L-mCherry(dup) in the virus stock can be demonstrated by PCR. The viruses that still retain the duplication are also expected to exhibit a delay in mCherry expression, but should still be capable of generating recombinant genes without necessitating factory fusion. What kind of timing characterizes this second class of mCherry expression kinetics?

**Fig 5:**
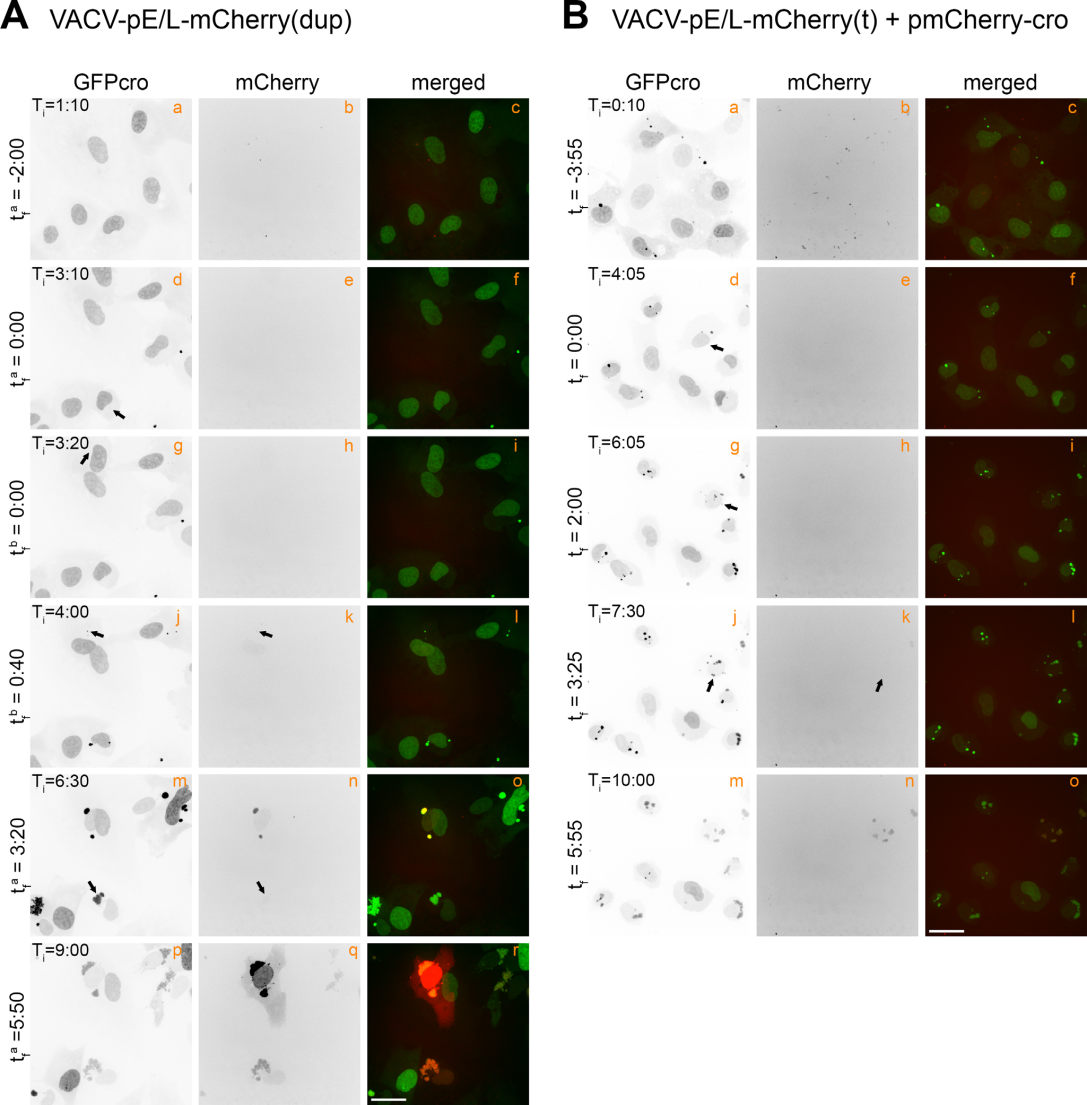
Timing of intraviral and virus-by-plasmid recombination events. (A) EGFPcro BSC-40 cells were infected with VACV-pE/L-mCherry(dup) at MOI = 0.5 to favor infections with a single particle. Two different cells are tracked here from the time of factory development: one infected by an actively recombining virus (t_f_^a^ = 0:00, panel d), and another presumed to be infected with a “pre-recombined” virus (t_f_^b^ = 0:00, panel g). The appearance of mCherry expression in the cell infected with the pre-recombined virus (t_f_^b^ = 0:40, panel k) mimics that seen in cells infected with the pE/L-mCherry-cro virus (Fig 2A, t_f_ = 0:35), while the actively recombining virus produces a mCherry-cro signal late in infection (t_f_^a^ = 3:20, panel n). (B) EGFPcro BSC-40 cells were transfected with linearized pmCherry-cro plasmid DNA 4 hours prior to infecting with VACV-pE/L-mCherry(t) at MOI = 5. Images were collected immediately after initiating the infection and the appearance of new EGFPcro labeled DNA was used to track factory development (t_f_ = 0:00; T_i_ = 4:05) while mCherry fluorescence was used to detect plasmid-by-virus recombination. Images were collected every 5 minutes up to 10 h post-infection and assembled into time-lapse movies (see S7 Video and S8 Video). The scale bar = 25 μm.

**Fig 6:**
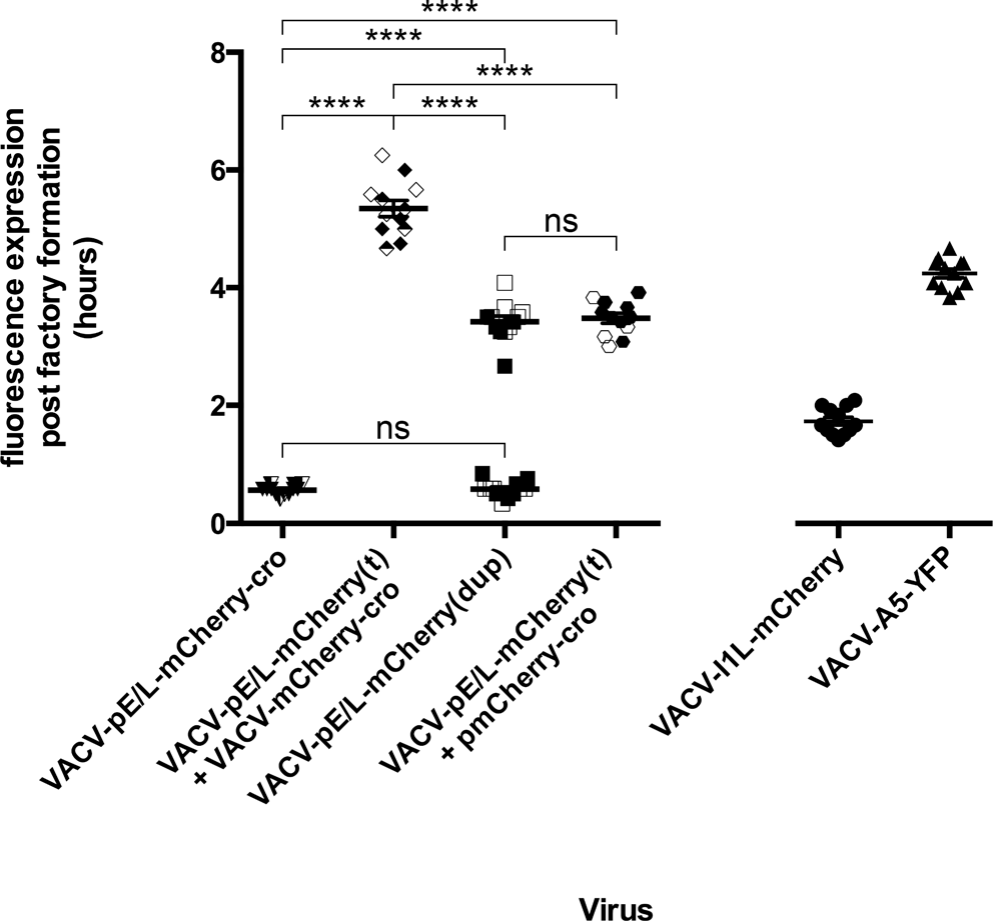
Summary of different reporter protein expression kinetics. The plot shows when a mCherry-cro signal is first detected relative to the time when a factory is first detected in that cell (t_f_). Each imaging experiment was repeated 3 times, and 4/10 fields in each experiment were analyzed in detail, to produce the 12 data points per infection that are shown here. The data show that the appearance of a mCherry-cro signal is significantly (****, p <0.001) delayed in cells co-infected with pE/L-mCherry(t) and mCherry-cro viruses, compared to any other kind of infection. Note that pE/L-mCherry(dup) infections show two patterns of gene expression, some virus produce an mCherry signal shortly after factories are detected, and others produce a signal delayed by ~3 h. The VACV-pE/L-mCherry(t) + pmCherry-cro experiment refers to cells where a promoterless mCherry-cro plasmid was transfected into cells infected with VACV-pE/L-mCherry(t). Note that only a single experiment was used to produce the 12 data points collected for the I1L-mCherry and A5-YFP infections, these serve as timing reference points for post-replicative and late VACV genes, respectively.

BSC-40 EGFP-cro cells were infected with pE/L-mCherry(dup) virus at MOI = 0.5, which according to a Poisson distribution maximized the chance (~90%) that each cell was infected with just one of the two predicted kinds of viruses. As expected we observed two distinct populations of viruses expressing mCherry. The first cluster of virus-infected cells exhibited mCherry expression kinetics identical to those previously exhibited by the pE/L-mCherry-cro control virus; t_f_^b^ = 0:40 (Fig 5A, panel k) *versus* t_f_ = 0:35 (Fig 2A, panel h). In contrast a second cluster of virus-infected cells exhibited mCherry expression kinetics significantly different from any seen previously. In these cells a mCherry signal was not detected until t_f_^a^ = 3:20 (Fig 5A, panel n) and the level of expression was markedly lower. To compare the two distinct populations seen in cells infected with partially duplicated virus, with other viruses, we measured the timing of mCherry expression relative to the first appearance of the factories in these cells. Twelve data points were collected for each virus as well as 12 of each kinetic class in pE/L-mCherry(dup) infected cells. These results, along with the data from Fig 2 and Fig 3, are summarized in Fig 6. Although one cannot determine with certainty when the “late” class of recombinants are being produced in cells infected with the pE/L-mCherry(dup) virus, it is apparent that these recombinants are already assembled by the time the associated late promoter is activated.

We also tested a second approach for measuring recombination timing and capacity. This method is based upon the observation that any DNA transfected into VACV-infected cells is replicated [28] within the virus factories [29]. This process is expected to create a large pool of substrate available for plasmid-by-virus recombination and in intimate contact with replicating virus genomes. In this experiment the BSC-40 EGFP-cro cells were first transfected with a plasmid encoding a promoterless copy of the mCherry-cro open reading frame (Fig 1A), 4 h prior to infection with pE/L-mCherry(t) virus at a MOI = 5. A complicating factor is that the transfected DNA is also stained with EGFP-cro (Fig 5B, panel b; S8 Video), but while these structures look superficially like virus factories, they are seen at time zero. The timing for the appearance of true virus factories (t_f_ = 0:00) was most accurately determined by tracking growing factories backwards to their initiating point. Interestingly, recombinant mCherry was detected at t_f_ = 3:25 (Fig 5B, panel k) a time essentially identical to that exhibited by the “late” class of recombinants formed in cells infected with the pE/L-mCherry(dup) virus (Fig 6).

Collectively, these experiments show that as long as there are no other physical impediments to recombination, a newly assembled recombinant gene under regulation of a VACV late promoter can be detected as soon as the promoter is activated. However, when the recombining elements are located *in trans*, on different viruses, the formation of a recombinant gene is further significantly delayed and well beyond the time point when the late reporter gene promoter is shown to become active. The implication is that this class of recombinant viruses is not assembled or matured until very late in infection.

### Recombinant frequency

**Fig 7:**
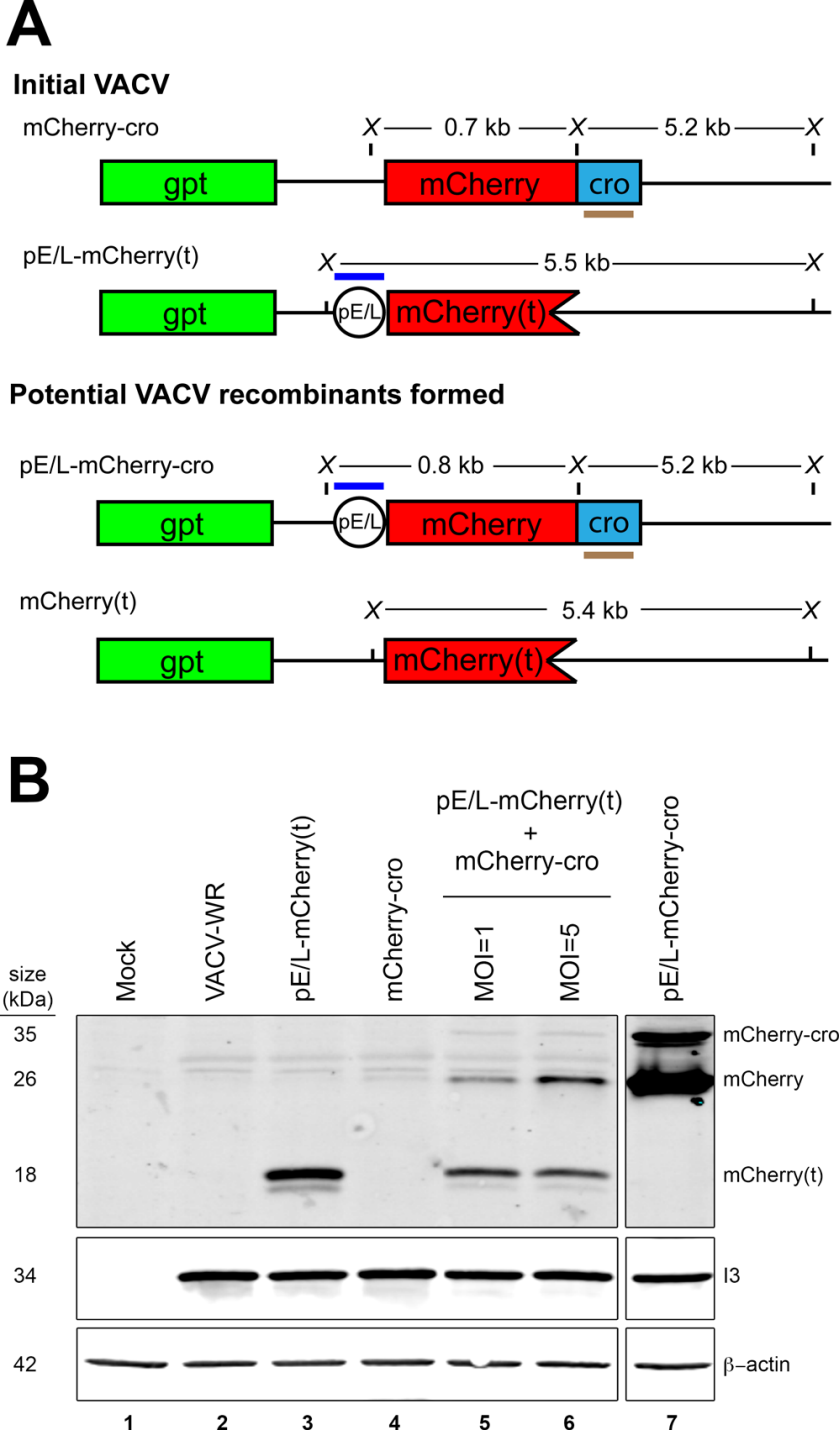
Quantifying the recombinants formed in co-infected cells. (A) The panel shows the two recombinant viruses that could be formed in cells co-infected with pE/L-mCherry(t) and mCherry-cro viruses. Also shown are the diagnostic restriction fragments that would be produced following *Xho*I digestion and the probes used for Southern blots (see Fig 8B). (B) Western blot analysis of proteins extracted from VACV-infected cells. BSC-40 cells were infected with the indicated viruses at a MOI = 5 (unless otherwise noted), harvested 24 h post-infection, and western blotted to detect recombinant mCherry-cro protein. A VACV gene product (I3) served as a marker of infection, and β-actin as a loading control. Note that both mCherry and mCherry-cro proteins are detected in cells infected with the control pE/L-mCherry-cro virus, suggesting that either could probably serve as a marker of recombination.

One cannot determine a recombinant frequency using purely optical methods, nor do these methods provide direct evidence of recombinant gene formation. A combination of western blotting, Southern blotting, and plaque counts, were used to measure these parameters in cells co-infected with pE/L-mCherry(t) and mCherry-cro viruses (Fig 7A). BSC-40 cells were separately infected, or co-infected, for 24 h with viruses encoding the truncated [pE/L-mCherry(t)] and/or promoterless (mCherry-cro) fluorescent proteins. Whole-cell lysates were then fractionated and western blotted to detect mCherry antigens (Fig 7B). An ~18 kDa N-terminal fragment was detected in cells infected with just the pE/L-mCherry(t) virus and lesser amounts of the same parental peptide were detected in cells co-infected with the two viruses (Fig 7B, lanes 3 and 5). Most critically, two recombinant peptides were detected in the co-infected cells and migrating at positions characteristic of mCherry (~26 kDa) and mCherry-cro (~35 kDa) proteins (Fig 7A, lanes 5–7). Judging by the control, both proteins are expressed by a recombinant pE/L-mCherry-cro virus (Fig 7B, lane 7). Proportionately more of the recombinant mCherry peptide, relative to the parental mCherry(t) peptide was also detected in cells co-infected at MOI = 5 versus MOI = 1 (Fig 7B, lanes 5 and 6).

We also used plaque assays to measure the fraction of recombinant viruses formed during a single round of infection. BSC-40 cells were co-infected with the pE/L-mCherry(t) and mCherry-cro viruses, at a combined MOI = 5, cultured overnight, and the progeny recovered by freeze-thaw 24 h post-infection. The viruses were then plated on BSC-40 cells and counted to determine the proportion of plaques exhibiting any degree of mCherry-positivity. The experiment was repeated three times and we detected 12 ± 1% red fluorescent recombinant plaques.

**Fig 8:**
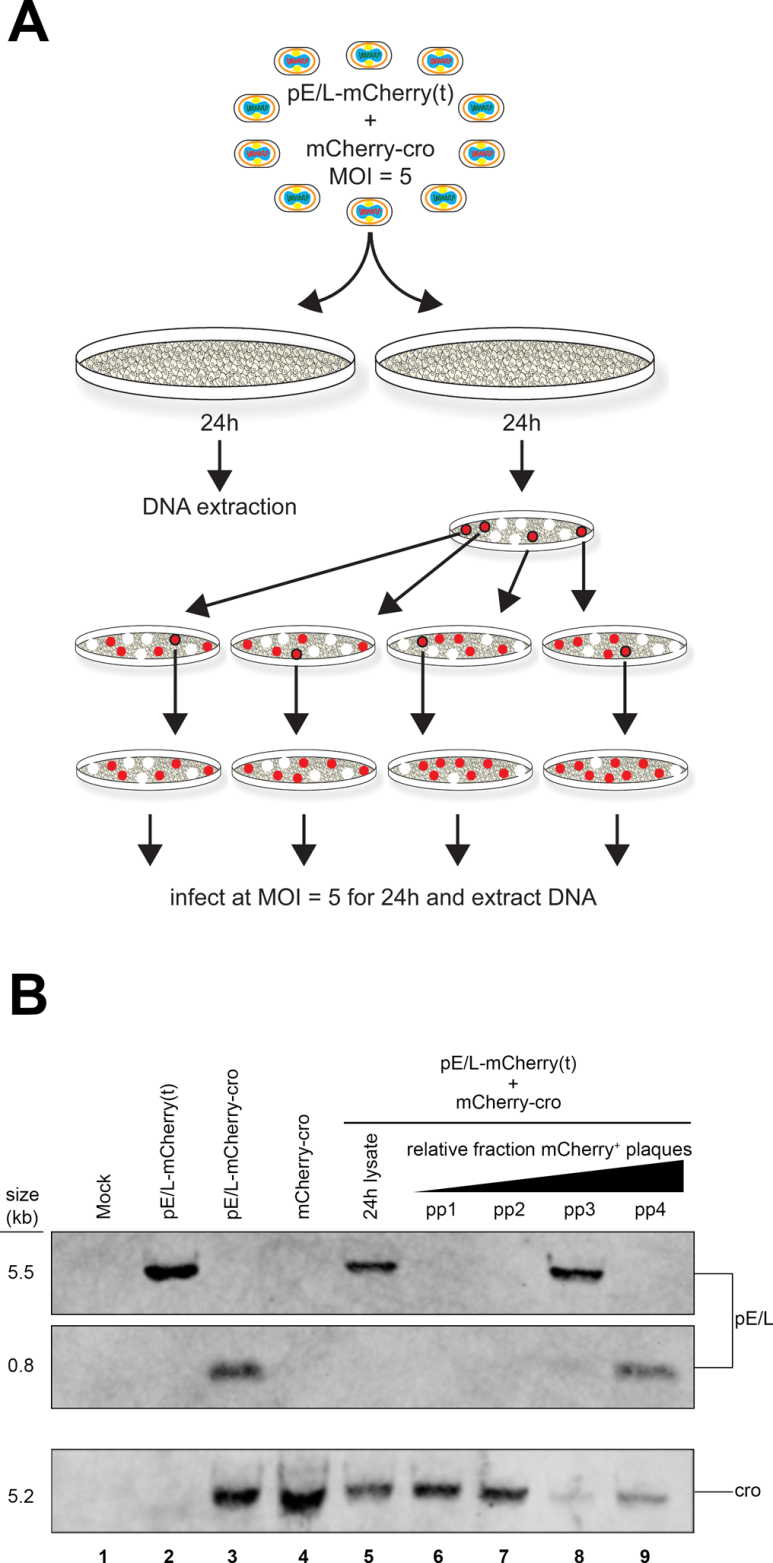
Southern blot analysis of recombinants. BSC-40 cells were infected (or co-infected) with the different indicated viruses, and Southern blotted to detect recombinants. (A) The scheme used to collect samples for Southern blotting. Some DNA was extracted directly from virus-infected cells 24 h after infection. Alternatively, the viruses were plated, red fluorescent plaques subjected to two rounds of plaque purification, and the virus expanded to produce sufficient DNA for Southern blots. (B) Southern blot analysis of virus DNAs. DNA was extracted from virus-infected BSC-40 cells, as in panel (A), digested with *Xho*I endonuclease, and blotted using biotin-labeled pE/L and cro probes (Fig 7A). A novel 0.8 kbp DNA fragment is diagnostic for recombinants (and is also found in the pE/L-mCherry-cro control virus), but this 0.8 kbp fragment is only detected after the recombinant viruses are subjected to several additional rounds of plaque purification.

Unfortunately this approach greatly overestimates the true recombinant frequency as was subsequently illustrated by the difficulties we had trying to detect recombinant genomes by Southern blotting. Two DNA probes were prepared that targeted either the pE/L poxvirus promoter, or sequences encoding the cro peptide (Fig 7A). The pE/L probe should detect a 5.5 kbp DNA fragment encoded by a parental virus [pE/L-mCherry(t)] and a 0.8 kbp fragment diagnostic for the recombinant virus, while the cro DNA probe is expected to detect 5.2 kbp DNA fragments encoded by the other parent (mCherry-cro) and by recombinant viruses (Fig 7A). These hybridization patterns were confirmed when total cellular DNA was extracted and Southern blotted from cells infected with either of the two parental viruses (Fig 8B, lanes 2 and 4), or with a virus duplicating the anticipated recombinant (pE/L-mCherry-cro; Fig 8B, lane 3). However, when DNA was extracted at 24 h post-infection from cells co-infected with the pE/L-mCherry(t) and mCherry-cro viruses, at a combined MOI = 5, we were unable to detect the 0.8 kbp fragment that is diagnostic for recombinant viruses (Fig 8B, lane 5). Further rounds of plaque purification showed that the mixture of viruses recovered from cells co-infected with the pE/L-mCherry(t) and mCherry-cro viruses do contain recombinant viruses, and these can be detected by Southern blotting. We picked four different red fluorescent plaques, performed one more round of plaque purification (again selecting for MPA-resistant and fluorescent viruses), grew up small stocks under MPA selection, and Southern blotted the DNA from these viruses. Some of the partly purified viruses now exhibited the 0.8 kbp restriction fragment diagnostic for a recombinant (Fig 7B, lanes 8 and 9), although two rounds of plaque purification were clearly still not sufficient to generate pure stocks of recombinants.

The challenge with these particular viruses is that it is hard to identify plaques formed by pure recombinants. We noticed that the plaques formed by viruses recovered from cells co-infected with the pE/L-mCherry(t) and mCherry-cro viruses exhibited a variable degree of red fluorescence. When we counted only plaques qualitatively exhibiting a high level of fluorescence, comparable to authentic pE/L-mCherry-cro recombinants, the recombinant frequency dropped to 1.9 ± 0.6% and suggested that many of the “recombinant” plaques might have been composed of a mix of parental viruses that produced recombinants subsequent to plating. The conclusion is that the propensity of VACV to form mixed plaques is a confounding factor, one that must be considered when attempting to measure recombinant frequencies using only plaque assays.

**Fig 9:**
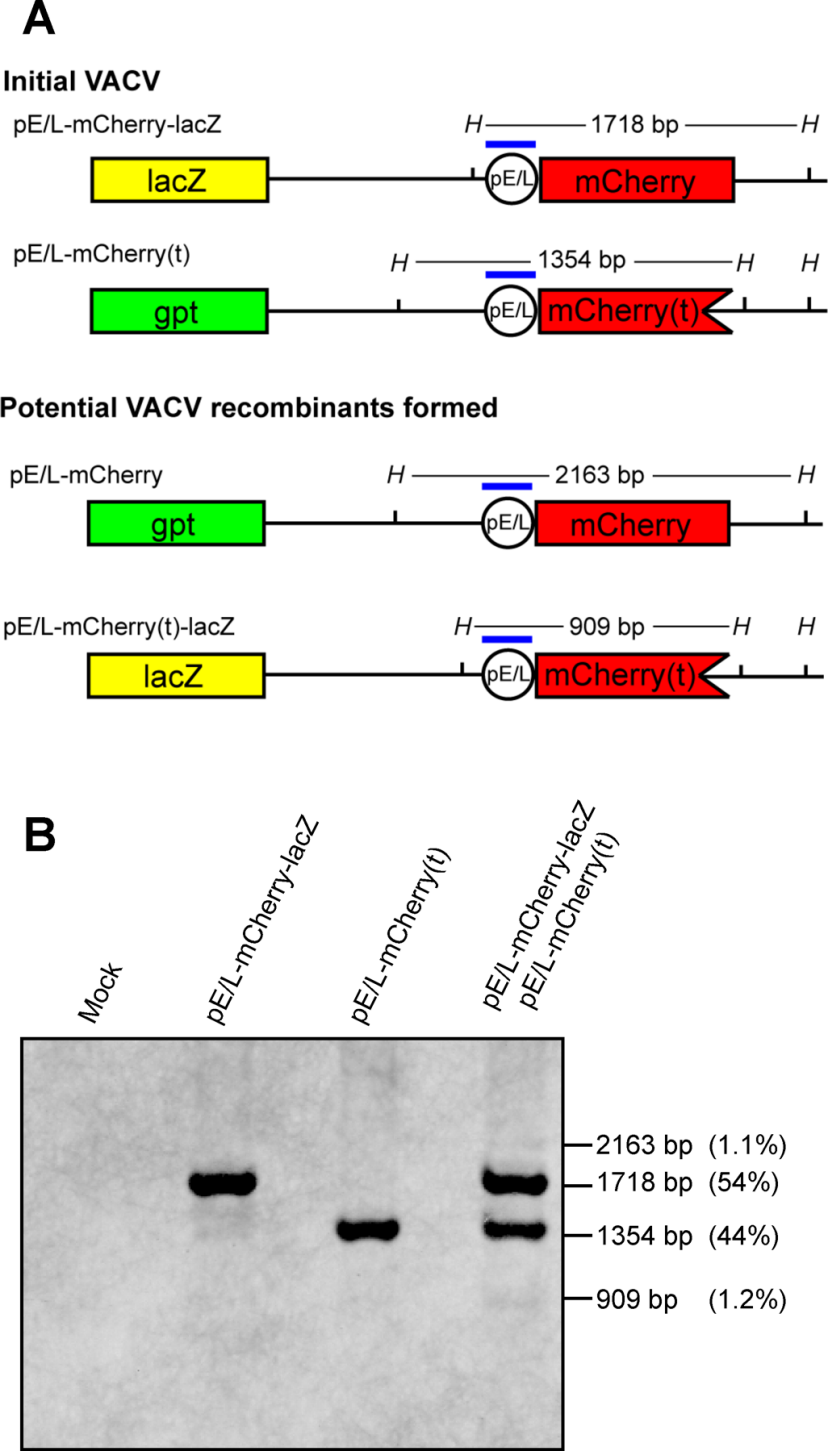
Recombination between pE/L-mCherry(t) and pE/L-mCherry-lacZ viruses. (A) The figure shows the two parent viruses, the predicted recombinants, and the *Hin*dIII fragments that should be detected by the pE/L oligonucleotide probe (blue bar). (B) Southern blot analysis of cells co-infected with the pE/L-mCherry(t) and pE/L-mCherry-lacZ viruses. BSC-40 cells were infected with each of the parental viruses at MOI = 5, or co-infected with the two viruses at a combined MOI = 5, and the DNA was extracted 24 h post-infection. The samples were then Southern blotted using a biotin-labeled probe. Although faint, two bands at 2.2 and 0.9 kbp are seen that indicate the presence of recombinant genomes. Collectively they comprise about 2% of the DNA.

Because of these concerns, we repeated the experiment using a different pair of viruses. One was the pE/L-mCherry(t) virus used in the preceding study, which also encodes the *gpt* marker that was used in virus construction [more properly it should be labeled pE/L-mCherry(t)-*gpt*]. The second encodes a *LacZ* marker replacing the *gpt* locus and expresses mCherry protein (pE/L-mCherry-*lacZ*). These viruses exhibit phenotypes of being either mCherry^-^ LacZ^-^ or mCherry^+^ LacZ^+^ and share a comparable amount (0.5 kbp) of homology with the preceding crosses, spanning the mCherry locus (Fig 9A). This strategy also eliminated the cro peptide, which seemed to have deleterious effects on viral replication as observed in the viral growth curves (S1 Fig). We infected BSC-40 cells with the two viruses either separately or together at MOI = 5, for 24 h, harvested total cellular DNA, and performed a Southern blot analysis using a probe specific for the synthetic poxvirus E/L promoter (Fig 9A). In parallel we plated the progeny virus on BSC-40 cells in the absence of selection and scored them using fluorescence microscopy followed by X-gal staining. Similar to the previous assay, we only scored mCherry^+^ plaques exhibiting a high level of red fluorescence. Using this approach we could accurately differentiate between viruses clearly exhibiting the parental (mCherry^-^ LacZ^-^ or mCherry^+^ LacZ^+^) *versus* recombinant (mCherry^+^ LacZ^-^ or mCherry^-^ LacZ^+^) phenotypes, since a mCherry^+^ LacZ^-^ recombinant is not easily confused with a mCherry^+^ LacZ^+^ parent. The Southern blotting detected a small fraction of recombinant genomes exhibiting novel 2.2 kbp and 0.9 kbp restriction fragments. These comprised 1.1% and 1.2% of the total viral DNA (Fig 9B, lane 4). Although this represented a small proportion of recombinants, in this experiment the numbers were close to those determined by plaque assays. The virus stocks isolated at 24 h post-infection contained 2.5% ± 0.6% and 2.8% ± 0.6% of the mCherry^+^ LacZ^-^ and mCherry^-^ LacZ^+^ recombinants, respectively, between four independent experiments. Given the good agreement between Southern blotting and plaque assays in this second experiment, and considering that the extent of homology was essentially the same in the two different types of crosses, we concluded that the events detected optically at the cellular level are probably associated with production of about 1–3% recombinants.

### Factories formed through fusion events retain internal ER boundaries

**Fig 10:**
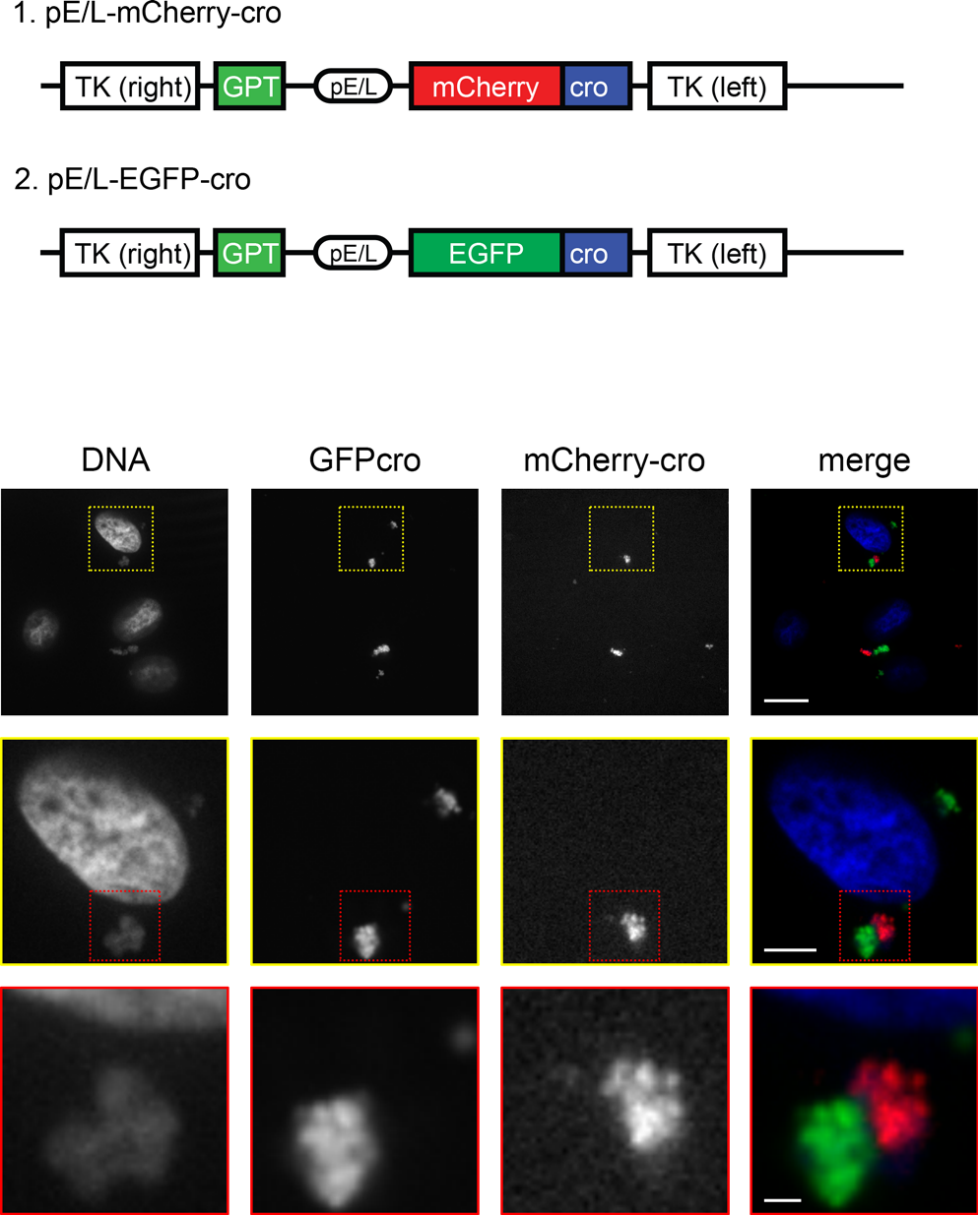
Maintenance of factory boundaries at an early stage of co-infection. BSC-40 cells were co-infected with pE/L-mCherry-cro and pE/L-EGFP-cro viruses (schematics, top) for 4 h and then fixed and stained with DAPI to also detect virus and cell DNA. The images shown here are taken from a single Z-stack showing closely associated factories, one labeled with EGFP-cro and the other mCherry-cro. The images were collected with a spinning disk microscope at 60× magnification. See S9 Video for an alternative view of the image. The scale bars are 15 μm (top), 5 μm (middle), and 1 μm (bottom panel).

These results still leave unanswered questions relating to why recombinant gene products are not detected until very late in infection and why such low recombination frequencies are detected when the genes are located *in trans*. One clue was provided by a different kind of experiment, one suggested by the earlier work of Katsafanas and Moss [30]. In addition to the pE/L-mCherry-cro virus (Fig 1), we had also previously constructed a virus encoding EGFP instead of mCherry protein [pE/L-EGFP-cro (Fig 10)]. Interestingly, we observed that some of the factories seen in cells co-infected with the mCherry-cro and EGFP-cro viruses were uniformly stained with mCherry protein, while other factories in the same cell were tagged with the EGFP protein (Fig 10; S9 Video). This showed that when the reporter protein is virus encoded it is not always freely diffusible. A possible explanation is that the bounding membranes that have been seen by electron microscopy [26] might be sufficiently contiguous as to limit protein movement between the factories originating as co-infecting viruses. These membranes are proposed to derive from the endoplasmic reticulum [26, 31], and if they were mostly intact then the DNA-binding proteins being synthesized on ER-associated ribosomes, might preferentially relocate to DNA binding sites located on the same side of the ER membrane. This then led us to wonder whether these ER boundaries might also continue to segregate the enclosed viroplasm, even after the factories have fused into larger assemblages.

**Fig 11:**
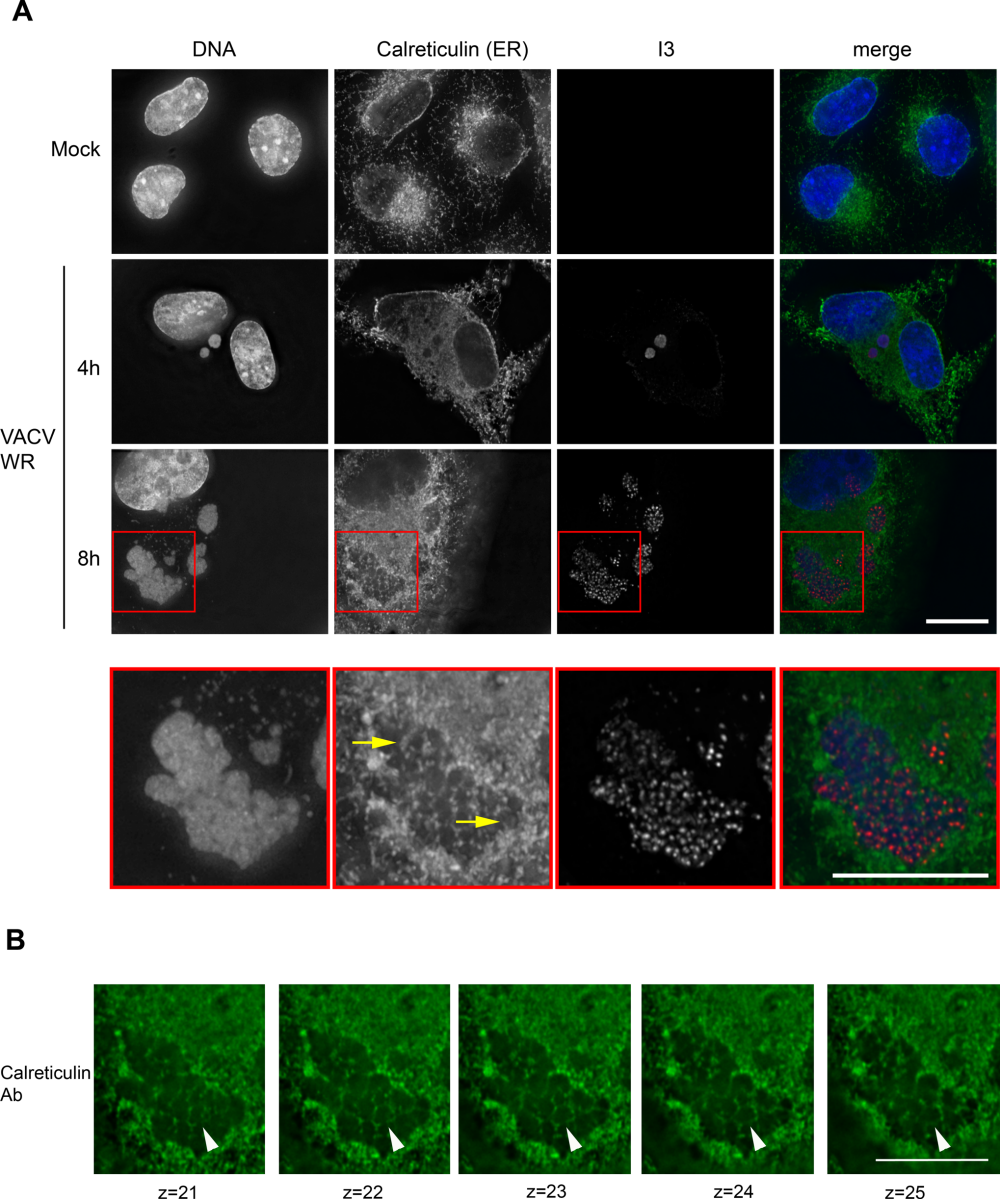
Large late viral factories enclose internal ER membranes. (A) BSC-40 cells were infected with VACV strain WR for 4 h (middle panel) or 8 h (third panel) and then fixed and stained to detect DNA (DAPI), the ER membrane marker calreticulin, and the viral I3 protein. The images in Panel A show a projection of the Z-stacks. At early time points (4 h post-infection) one sees no calreticulin staining within the small early virus factories, although it is widely distributed throughout the cytoplasm. At later times, however, calreticulin-positive ER membranes appear to traverse these large late viral factories. This feature is more readily seen in an enlargement of the factory area, shown in the bottom row. (B) The same region of the image was separated into the component Z-stacks, these serial sections show the ER membranes extending downwards, through the factory. See S10 Video for an alternative view of the image. These images were acquired using an Olympus IX-71 inverted microscope at 60× magnification and deconvolved using Softworx software (GE Healthcare). The scale bar = 15 μm and each Z-stack spans 200 nm.

To examine this question we used fluorescence microscopy to image the distribution of ER membranes in VACV factories at different times in the infection cycle. An antibody targeting the ER marker calreticulin [32] was used to track the distribution of ER membranes. At an early time point (4 h), the calreticulin marker was distributed throughout the cytoplasm and also seen excluded from regions containing the virus DNA (Fig 11). It was not seen within the small factories at this stage in their development. However, later in the infection cycle (8 h), when many factory fusion events would have been expected to occur, the ER marker was seen forming a reticulated pattern within the now larger assemblages (Fig 11). The calreticulin stain could be traced through the optical sections, outlining a number of what look like subdomains within the larger structures. This can be traced through a series of separate image stacks spanning >1 μm (Fig 11B). Elsewhere in this particular image one can see less intimately fused factories, clearly separated by opposed bounding membranes (Fig 11A, 8 h). We interpret these images to mean that even though VACV factories are seen fusing during the course of infection, this process would not necessarily lead to DNA mixing, due to the continued presence of one or more of the original bounding ER membranes. These membranes are disassembled as virus assembly starts late in infection [26], and perhaps only then can the DNAs of co-infecting viruses mix well enough to permit recombination *in trans*.

## Discussion

These studies provide insights into when recombinant genes can be formed during VACV replication and how that process is affected by the arrangement of the recombining fragments *in cis* (i.e. on the same genome), or *in trans* (on different genomes). The technology is somewhat constrained by the limits imposed by the kinetics of virus promoter activation, nevertheless some important general features of poxvirus recombination are illustrated by these studies.

These experiments employed EGFP and mCherry fluorescent proteins fused to a phage lambda cro DNA binding domain. The cell-encoded EGFP-cro protein permitted tracking of replicating virus particles, while modified forms of virus-encoded mCherry-cro protein permit detection of gene rearrangements. Controls showed that a mCherry-cro signal is detected very shortly after new factories are first tagged with EGFP-cro in cells infected with pE/L-mCherry-cro virus. The ~35 min gap from the appearance of the first factories would likely be related to the time needed to fold the newly expressed mCherry protein (~15 min) and to concentrate it enough to see as DNA is exposed in newly uncoated viruses. Like the EGFP-cro protein that we have previously studied, mCherry-cro associated with both factories and the nucleus, but exhibits a preference for VACV DNA (S3 Video).

A different pattern of mCherry-cro expression was seen in cells infected with the pE/L-mCherry(dup) viral construct. Viruses encoding partly duplicated DNA segments, such as this one, are unstable unless one maintains selection for the parental virus [33]. When cells were infected at low multiplicities of infection with these viruses, one can see two different patterns of mCherry expression with timing characteristic of either early or late VACV promoters (Fig 6). The first class of events, which express mCherry very shortly after the first factories are detected, presumably reflect pre-existing recombinants that were formed during preparation of the virus stock. The same mCherry expression kinetics was seen as in cells infected with the pE/L-mCherry-cro control virus.

More interesting is the second class of recombination events seen in cells infected with the pE/L-mCherry(dup) virus, where mCherry is not detected until the activation of late promoters (~3:20 after factories are first detected). This presumably reflects the transcription and translation of recombinant genes formed during the preceding period of DNA replication. We have not tried to establish further the exact timing of such events, although one could probably narrow it down more using promoters based upon those regulating post-replicative genes. Most probably these reactions take place throughout the period of DNA replication when the enzymes needed to catalyze both viral replication and recombination are present. The same timing of appearance of a late mCherry signal (t_f_ = 3:25) is also seen in cells transfected with a plasmid encoding a promoterless copy of the mCherry-cro gene and infected with pE/L-mCherry(t) virus. A feature common to both situations is that all of the interacting genetic components would be mixed closely together within the factories and from an early stage in virus development. In the case of the pE/L-mCherry(dup) this is because of the physical linkage of the recombining elements, in the case of the transfected cells it is because non-specific DNA replication of transfected DNAs [28] takes place in viral factories [29].

Quite different expression kinetics were seen in cells co-infected with the pE/L-mCherry(t) and mCherry-cro viruses. In this case a recombinant can only be assembled through an exchange between gene fragments located *in trans* on different genomes and through a reaction requiring second order reaction kinetics. Given that each factory is understood to begin as a single infecting particle [25, 30, 34], the fusion of different factories bearing different VACV genotypes would seem to be required in advance of any recombinant forming reactions. The time it takes to observe factory fusion is a function of the multiplicity of infection, although even at high multiplicities of infection a small portion of viral factories never fuse [25]. In these current studies, we saw varying times to fusion, but in the example shown in Fig 2B (panel g), we detected the first mergers very shortly after factories first appeared (t_f_ = 0:35) and these were followed by further aggregation of the different virus factories into larger assemblies over the next few hours. Not all fusion events would necessarily aggregate viruses comprising the two different genotypes, but over the long course of infection at a combined MOI = 5 at least some co-mingling of different virus genotypes is bound to occur.

Interestingly, even though fusion events were observed throughout the period of virus replication in co-infected cells, it wasn’t until an average t_f_ = 5:20 that the first signs of recombinant mCherry-cro protein were detected (Fig 6, S4 Video). This is two hours after the late class of recombinants were detected in cells infected with the pE/L-mCherry(dup) virus and roughly coincident with the point when the factories started to exhibit a more diffuse appearance. As the mCherry signal appeared, it showed up simultaneously in all the factories, rendering it impossible to determine if it originated from a particular source. These observations suggest that while factory fusion would seem to be needed to mix the genotypes essential for recombination *in trans*, this alone is not sufficient to create the environment needed to produce recombinants. Otherwise one would expect to have seen at least some mCherry signals appearing as soon as the E/L promoter was activated in co-infected cells and around the same time as the late class of recombinants were detected in cells infected with the pE/L-mCherry(dup) virus. Also notable was the low intensity of the fluorescent signal. This could be related to reduced levels of transcription and translation by that time point. However, Southern blotting and plaque assays also detected about 1–3% recombinant genomes and virus plaques, reflective of the low level of recombinant protein expression.

These observations suggest that recombination between co-infecting VACV is restricted by more factors than just factory fusion. One thing that we had noted previously, using fluorescence *in situ* hybridization (FISH), was that even after the factories have merged, a large portion of the DNA encoding the two different genotypes remained segregated within the larger structures [25]. Moreover we (Fig 10), and others [30], see some evidence that virus-encoded proteins are not always freely diffusible between different factories. In this regard J. Locker’s previous studies concerning the ultrastructure of VACV replication sites become highly relevant [26]. Her electron micrographs showed that rapidly growing viral factories are nearly completely (80–85%) bounded by membranes from the endoplasmic reticulum. Moreover, these extensive bounding membranes are disassembled as immature virions begin to form. One might expect that were factories to fuse during the course of infection, only along the boundary between two different fused factories would there be any initial opportunity for DNA to mix. However, such mixing would be greatly limited if what once comprised that boundary was “fenced in” by stable membranes and the virus DNA perhaps further constrained by the DNA and membrane binding protein E8 [35]. This is precisely what is seen in larger late factories prior to their dissolution, the ER marker calreticulin enclosing the viroplasm within different subdomains (Fig 11A). Although the VACV factories are fusing, they appear to retain what we presume are the original bounding membranes. It then starts to become clear why a recombinant mCherry signal doesn’t start to appear until after the well-demarked and larger late factories have started to break down into more diffuse structures (Fig 11B). Presumably only then are virus DNAs finally able to mix freely. Of course by this time point, the capacity to process recombination intermediates into mature and intact DNA duplexes would also start to go into decline as VACV transitions from the replication phase into the assembly phase. The cumulative effect would be to limit the amount of recombinants formed in co-infected cells.

These observations do explain one of the more confusing features of poxvirus biology, which is that transfected molecules exhibit extraordinarily high levels of recombination, while viruses do not. For example, one can detect high levels of recombinant formation among DNAs transfected into Shope fibroma virus and VACV infected cells, with linkage lost beyond 300–500 bp in some experiments using Shope fibroma virus infected cells [13, 36]. In contrast, the current study detected only 1–3% recombinants formed in a single co-infection and this is in rough agreement with data from genome sequencing [~1 exchange per 10 kbp [12]]. The simplest explanation is that poxvirus recombination systems are very active, but transfected DNA also mixes well and is not subjected to the same constraints that viruses are.

In conclusion, these studies show that where DNAs can mix intimately, recombinant viruses are detected as soon as the promoters and reporter genes permit their detection. However, two co-infecting VACV seem to face several impediments to recombination, *in trans*, that collectively delay and reduce the yield of recombinant viruses. Most importantly, even though the factories formed by different co-infecting viruses can fuse throughout the infection cycle, the bounding ER membranes would probably continue to limit complementation and partially isolate the different genotypes as they are being replicated. The DNA cannot mix, and recombinants cannot form, until these structures are disassembled. However, by that point the systems that might catalyze recombination are in competition with processes associated with virus assembly, greatly reducing the capacity to produce recombinants. This intriguing biology would tend to be a stabilizing factor in virus evolution, and disfavor accumulation of defective interfering particles in culture, and it raises interesting questions regarding how it might have affected the evolutionary trajectory of such apparently ancient [37] and successful viral pathogens.

## Materials and Methods

### Cells, viruses, and other reagents

African green monkey kidney epithelial cells (BSC-40) were purchased from the American type culture collection (ATCC) and grown in modified Eagle’s medium supplemented with non-essential amino acids, L-glutamine, antibiotics/antimycotics, and 5% fetal bovine serum, which were all purchased from Thermo Fisher Scientific. BSC-40 cell lines constitutively expressing the bacteriophage lambda cro protein fused to either enhanced green fluorescent protein (EGFP-cro) or mCherry fluorescent protein (mCherry-cro), were prepared as previously described [25]. All of the recombinant viruses used in this study were derived from VACV strain Western Reserve, our stock was originally obtained from the ATCC. A virus encoding VACV A5 protein fused to yellow fluorescent protein (YFP) was obtained from Dr. B. Moss [30]. Growth curves used BSC-40 cells infected with virus at a multiplicity of infection (MOI) of 3. The virus were harvested at the indicated time point, released by freeze-thaw, diluted, and the yield determined by plaque assay on BSC-40 cells.

### Recombinant virus construction

The viruses were prepared by first cloning parts (or all) of a gene encoding mCherry fluorescent protein fused to the phage lambda cro DNA binding domain into plasmid pTM3 [38] and flanked by VACV thymidine kinase gene sequences. A detailed description of how each of the precursor plasmids was first assembled is provided as supplementary material (S1 Methods). To generate each virus, BSC-40 cells were first infected with VACV at a MOI of 3 for 2 h followed by transfection of the linearized recombinant plasmid using Lipofectamine 2000 (Invitrogen), and the recombinant viruses then isolated using modified Eagle’s medium supplemented with 25μg/mL mycophenolic acid, 15μg/mL hypoxanthine, and 250μg/mL xanthine (Sigma). The viruses were plaque purified at least three times and purified by centrifugation through a sucrose cushion [25]. Fig 1A illustrates the different viruses assembled for this study. Note that the virus called “pE/L-mCherry(dup)” is intrinsically unstable, no doubt due to recombination [33], but the duplication can be maintained by continued passage in media containing mycophenolic acid.

### Fluorescence microscopy

All of the live-cell imaging studies were performed using an Olympus IX-81 spinning-disc confocal microscope equipped with a heated cell chamber and providing a 5% CO_2_ atmosphere. Briefly, the cells were first cultured on optically clear 35 mm glass bottom dishes (Fluorodish, World Precision Instruments) and then infected with virus for 1 h at 4°C in serum-free MEM containing 10 mM HEPES pH 7.2–7.5. The inoculum was then replaced with warmed FluoroBrite Dulbecco’s modified Eagle’s media (Thermo Fisher Scientific) supplemented with 10 mM HEPES pH 7.2–7.5, nonessential amino acids, and 5% fetal bovine serum and incubated for another hour at 37°C. The dishes were sealed with Parafilm, and mounted on the 37°C microscope stage. For virus-by-plasmid recombination imaging, 4 h prior to initiating infection (as described above) 2μg of linearized plasmid DNA was transfected into EGFP-cro cells using Lipofectamine 2000. Image data were collected using a 40×/1.3-numerical aperture (NA) oil PlanApoN objective at 5-minute intervals using Volocity software (Perkin-Elmer). EGFP was detected using the fluorescein isothiocyanate (FITC) filter set and mCherry was detected using the red fluorescent protein (RFP) filter set. Ten separate fields of view were typically recorded in a given experiment.

For fixed-cell imaging, the cells were first seeded on glass cover slips in 24-well plates and infected for 1 h at 4°C in serum-free MEM supplemented with 10 mM HEPES pH 7.2–7.5. The inoculum was replaced with fresh warmed MEM supplemented with nonessential amino acids, L-glutamine, antibiotics/antimycotics, and 5% fetal bovine serum; and returned to the 37°C incubator until the desired time point was reached. The samples were fixed at 4°C overnight using 4% paraformaldehyde in phosphate-buffered saline (PBS), and then quenched with 0.1 M glycine for 30 min. The cells were permeabilized with PBS containing 0.1% Triton-X100 (PBS-T), counter-stained with 0.1 μg/mL 4’,6-diamidino-2-phenylindole (DAPI, Molecular Probes) in 50% (v/v) Odyssey blocking buffer (Li-Cor) in PBS, washed with PBS-T, washed again with PBS and mounted using Mowiol mounting medium (0.1 mg/ml Mowiol, 0.1 M PBS, pH 7.4, 25% glycerol, 2.4% triethylenediamine [DABCO]). Where indicated, an antibody recognizing calreticulin was used (Abcam AB2907) to visualize endoplasmic reticulin. The fixed cell images were acquired using a 60×/1.42 NA oil PlanApoN objective using DAPI, FITC, RFP, and CY5 filter sets.

### Recombinant frequency

Two approaches were used to determine the recombinant frequencies. In the first, BSC-40 cells were co-infected at a combined MOI = 5 with a 1:1 mix of mCherry-cro and pE/L-mCherry(t) viruses (Fig 1A). Fresh media was added after 1 h and the plates returned to the incubator overnight. The cells were harvested next day (24 h), by scraping them into the medium, the virus released by three rounds of freeze-thaw and plated at high dilution on BSC-40 cells in 6-well dishes. A Zeiss inverted fluorescence microscope was used to detect and count well-resolved mCherry-positive plaques using an RFP filter set and DIC optics. The plates were then stained with crystal violet to determine the total plaque count.

In a second experiment, BSC-40 cells were co-infected at a combined MOI = 5 with a 1:1 mix of pE/L-mCherry-*lacZ* (Fig 9) and pE/L-mCherry(t) viruses (Fig 1A). The viruses were cultured and plaqued, and the mCherry positive plaques were identified by fluorescence microscopy as described above. The plates were then stained with 5-bromo-4-chloro-3-indolyl-β-D-galactopyranoside to differentiate LacZ^+^ viruses from those bearing the guanosylphosphoribosyl transferase marker on the [pE/L-mCherry(t)] virus.

### Western blotting

BSC-40 cells were cultured in 10 cm dishes and then infected with virus as described above. Twenty four hours post infection, the cells were harvested into cold PBS, centrifuged at 1,000× *g* for 3 min and lysed on ice in radioimmunoprecipitation assay (RIPA) buffer (50mM Tris-HCl pH7.4, 150mM NaCl, 1mM EDTA, 1% NP-40, 0.25% Na-deoxycholate) containing 1× protease inhibitors (Roche). The samples were clarified by centrifugation, and boiled briefly in sample buffer (50mM Tris·HCl pH6.8, 3.7% sodium dodecyl sulfate, 0.6M β-mercaptoethanol, 1.5mM bromophenol blue, in 40% glycerol). The samples were size fractionated on 12 or 15% SDS-polyacrylamide gels, and then transferred to a nitrocellulose membrane (Thermo Fisher Scientific). The membranes were incubated overnight at 4°C with appropriate antibodies. These included mCherry (1:2,000 diluted rabbit polyclonal; Clontech), VACV I3 (1:5,000 diluted mouse monoclonal antibody [39]), VACV A34 (1:10,000 diluted rabbit polyclonal antibody; this laboratory), and/or β-actin (1:20,000 diluted mouse monoclonal antibody; Sigma). The membranes were subsequently exposed to a 1:20,000 diluted secondary antibodies bearing infrared dyes (goat-anti-rabbit 680 and goat-anti-mouse 800; Li-Cor) for 1 h at room temperature and imaged using a Li-Cor Odyssey scanner. Gel images were analyzed using FIJI [40].

### Southern blotting

DNA was purified from virus-infected BSC-40 cells by phenol/chloroform extraction and ethanol precipitation, digested with *Xho*I or *Hin*dIII (Fermentas), and size fractionated on 0.7% agarose gels. The DNA was denatured *in situ* in a solution containing 0.5M NaOH and 1.5M NaCl, transferred to a nylon membrane (Pall Corporation), and fixed by UV cross-linking. A biotin-containing cro-gene probe was prepared using two primers (5’-TGATGGAACAACGCATAA-3’ and 5’-TTATGCTGTTGTTTTTTTGTTAC-3’), biotin-16-dUTP (Roche), template DNA, and the PCR. A second probe was purchased from IDT as a biotin-labeled oligonucleotide (5’-biotin-AAAAATTGAAATTTTATTTTTTTTTTTTGGAATATAA-3’). It detects the synthetic early-late promoter driving mCherry gene expression. The probes were hybridized to the prepared membrane using ExpressHyb (Clontech), and detected using streptavidin conjugated to IRDye 800CW (Li-Cor) and an Odyssey Li-Cor scanner.

### Image data processing and analysis

Image data files were exported as either Volocity or Softworx files and then assembled into composite images using FIJI and Photoshop CS6 [40]. The images acquired in each experiment were subjected to the same scaling adjustments using only linear gamma factors. Labels were added to the video images using Camtasia 2.0. Greyscale images were prepared using Adobe Photoshop CS6 and GraphPad Prism v6 was used for statistical analyses.

## Supporting Information

S1 Methods Detailed methods. (DOCX)

S1 Fig Virus growth curves. BSC-40 cells were infected with the indicated viruses at a MOI = 3. Viruses were harvested at the indicated time points and titered on BSC-40 cells. The mean ± S.E.M. from three independent experiments are shown.
(TIF)

S2 Fig Quantification of factory fusion. The two upper panels show two consecutive fluorescence images spanning 5 min. Fluorescence intensities from virus factories in the highlighted cell (red box) were quantified using FIJI software. The particles designated B and C seemed to fuse into a single larger and brighter particle (B + C). The two lower panels show the distribution of fluorescence in the above images. Numbers below labeled virosomes indicate the mean fluorescence intensities.
(TIF)

S1 Video Live cell video showing EGFP-cro BSC-40 cells infected with mCherry-cro virus. Images were collected every 5 min up to 10 h post-infection and incorporated to produce the video.
(MP4)

S2 Video Live cell video showing EGFP-cro BSC-40 cells infected with pE/L-mCherry(t) virus. Images were collected every 5 min up to 10 h post-infection and incorporated to produce the video.
(MP4)

S3 Video Live cell video showing EGFP-cro BSC-40 cells infected with pE/L-mCherry-cro virus. Images were collected every 5 min up to 10 h post-infection and incorporated to produce the video. The arrows match the panels seen in Fig 2A, and mark the first factory formed in the cell of interest, first sign of mCherry-cro production, and the mCherry-cro seen in a viral factory late in infection.
(MP4)

S4 Video Live cell video showing EGFP-cro BSC-40 cells co-infected with pE/L-mCherry(t) and mCherry-cro viruses. The images were collected every 5 min up to 10 h post-infection and incorporated to produce the video. Arrows were added to match the panels seen in Fig 2B and show the initial factory formation in the cell of interest, two factories fusing into one brighter factory, first sign of mCherry-cro production, and mCherry-cro seen in a viral factory at late stages of infection.
(MP4)

S5 Video Live cell video showing EGFP-cro BSC-40 cells infected with I1L-mCherry virus. The images were collected every 5 min up to 10 h post-infection and incorporated to produce the video. Arrows were added to match the panels seen in Fig 3A and show the initial factory formation, first sign of I1-mCherry production, and mCherry seen in viral factories at late stages of infection.
(MP4)

S6 Video Live cell video of mCherry-cro BSC-40 cells infected with A5L-YFP virus. Images were collected every 5 min up to 10 h post-infection and incorporated to produce the video. Arrows were added to match the panels seen in Fig 3B and show the initial factory formation, first sign of newly produced A5-YFP at viral factories, and YFP tagged A5 core protein in viral factories at late stages of infection.
(MP4)

S7 Video Live cell video of EGFP-cro BSC-40 cells infected with pE/L-mCherry(dup) virus. Images were collected every 5 min up to 10 h post-infection and incorporated to produce the video. Arrows were added to match the panels seen in Fig 5A, tracking both a previously recombined virus (like the pE/L-mCherry-cro virus), and a virus undergoing intra-molecular recombination. The arrows denote the initial factory formations in the cells of interest, and first signs of mCherry-cro production in the two different populations.
(MP4)

S8 Video Live cell video of plasmid (pmCherry-cro) transfected EGFP-cro BSC-40 cells infected with pE/L-mCherry(t) virus. Images were collected every 5 min up to 10 h post-infection and used to produce the video. Unlike most of the imaging experiments, where we waited 1 h before starting to collect data, the imaging in this study was started immediately after infection. Arrows were added to match the panels seen in Fig 5B showing the transfected DNA, initial factory formation, first sign of mCherry-cro production, and mCherry-cro localized at viral factories at later stages of infection.
(MP4)

S9 Video Three-dimensional rendering of a BSC-40 cell co-infected with pE/L-mCherry-cro and pE/L-EGFP-cro viruses. The images were collected using 0.5 μm stacks and assembled into a 3-D model using Volocity 3D opacity. A single slice of this imaging experiment was presented in Fig 10.
(MP4)

S10 Video Translation through the Z-stacks in a large late VACV factory. Z Stacks #16 to 31 were combined to produce a video version of the data presented in Fig 11. DNA is stained with DAPI (blue), I3 in red, and the ER membrane marker calreticulin in green.
(MP4)

S1 References Supporting information references. (DOCX)
