# Supplementary material for: Live-Cell Imaging of Vaccinia Virus Recombination
Source: PLoS Pathog. 2016 Aug 15;12(8):e1005824. doi: 10.1371/journal.ppat.1005824 (PMC4985154; doi:10.1371/journal.ppat.1005824)
Supplement: S1 Methods — (DOCX) [file ppat.1005824.s001.docx]

**S1 Methods**

**Plasmid construction**. To create a mCherry-cro template DNA (for PCR), an EGFP-cro encoding plasmid [1] and pmCherry-C1 (Clontech [Cat No. 632524]) were digested with *Hin*dIII and *Bam*HI, the DNAs were gel purified, and ligated to produce plasmid pmCherry-cro-C1. Plasmid pTM3 encodes sequences homologous to the VACV thymidine kinase locus, flanking a multiple cloning site, and was obtained from B. Moss.

The PCR and primers 1F+2R (see below) were used to amplify the pE/L-mCherry-cro gene. The DNA was gel purified, cut with *Xho*I and *Eco*RI and cloned into *Xho*I- and *Eco*RI-cut pTM3 to create pTM3-pE/L-mCherry-cro. Plasmid pTM3-pE/L-mCherry(t) was assembled using a PCR fragment amplified using primers 1F+3R, that was then cut with *Xho*I and *Eco*RI, and cloned into *Xho*I- and *Eco*RI-cut pTM3. Plasmid pTM3-mCherry-cro was assembled using the same strategy, but using primers 4F+2R.

To create the plasmid encoding a partially duplicated mCherry-cro gene, a more complex strategy was needed. A PCR reaction, pmCherry-cro-C1, and primers 5F+6R were used to first synthesize a pE/L-mCherry(t) fragment. The pE/L-mCherry(t) DNA, and plasmid pTM3, were digested with *Eco*RI and *Spe*I, gel purified, and ligated to create the intermediate plasmid pTM3-pmCherry(dup)^1/2^. The downstream overlapping mCherry-cro gene fragment was synthesized using a PCR reaction, plasmid pmCherry-cro-C1, and primers 7F+8R. The PCR product and pTM3-mCherry(dup)^1/2^ were both digested with *Sph*I, gel purified, and the PCR fragment cloned into alkaline phosphatase treated *Sph*I-digested pTM3-mCherry(dup)^1/2^ to create plasmid pTM3-pmCherry(dup).

The PCR, primers 9F+2R, and plasmid EGFP-cro [1] were used to create a DNA bearing an EGFP-cro gene fused to a VACV E/L promoter. The PCR product was cloned into pCR2.1-TOPO, amplified again using the PCR and a M13 primer set (10F+11R) and cloned into pTM3 using *Pst*I and *Eco*RI. This created plasmid pTM3-pE/L-EGFP-cro.

**Other viruses.** VACV-I1L-mCherry was constructed using a plasmid synthesized by GeneArt (Thermo Fisher Scientific). The plasmid encoded the VACV I1L gene fused in-frame with a C-terminal mCherry gene and flanked by ~250 bp regions of homology. The linearized plasmid was transfected into VACV-infected BSC-40 cells, and mCherry positive recombinants isolated using 3 rounds of plaque purification.

VACV-pE/L-mCherry-*lacZ* was constructed by first digesting plasmid pE/L-mCherry-TOPO [2] with *Not*I to obtain the pE/L-mCherry fragment. The fragment was cloned into pSC66 [3], which contains regions of homology flanking J2R and a *lacZ* gene under the control of a VACV p7.5 promoter. The pE/L-mCherry-*lacZ* plasmid was transfected into VACV-infected BSC-40 cells and mCherry positive recombinants isolated using 3 rounds of plaque purification.

| **Primer #** | **Primer sequence (5’ to 3’)** |
| --- | --- |
| 1F | CGATCACTCTCGAGAAAAATTGAAATTTTATTTTTTTTTTT­TGGAATATAAATGGTGAGCAAGGGCGAGG |
| 2R | CTAGCTGAGAATTCTTATGCTGTTGTTTTTTTGTTAC |
| 3R | CTAGCTGAGAATTCCTACTGCTTGATCTCGCCCTTCAGG |
| 4F | CGATCACTCTCGAGATGGTGAGCAAGGGCGAGG |
| 5F | CGATCACTGAATTCAAAAATTGAAATTTTATTTTTTTTTTT-TGGAATATAAATGGTGAGCAAGGGCGAGG |
| 6R | CTAGCTAACTAGTCTACTGCTTGATCTCGCCCTTCAGG |
| 7F | CGATCACTGCATGCGTGAGCAAGGGCGAGGAGG |
| 8R | CTAGCTGAGCATGCTTATGCTGGTGTTTTTTTGTTAC |
| 9F | CGATCACTCTGCAGAAAAATTGAAATTTTATTTTTTTTTTT-TGGAATATAAATGGTGAGCAAGGGCGAGG |
| 10F | GTAAAACGACGGCCAG |
| 11R | CAGGAAACAGCTATGAC |
