# Supplementary figures and images for: Live-Cell Imaging of Vaccinia Virus Recombination
Source: PLoS Pathog. 2016 Aug 15;12(8):e1005824. doi: 10.1371/journal.ppat.1005824 (PMC4985154; doi:10.1371/journal.ppat.1005824)

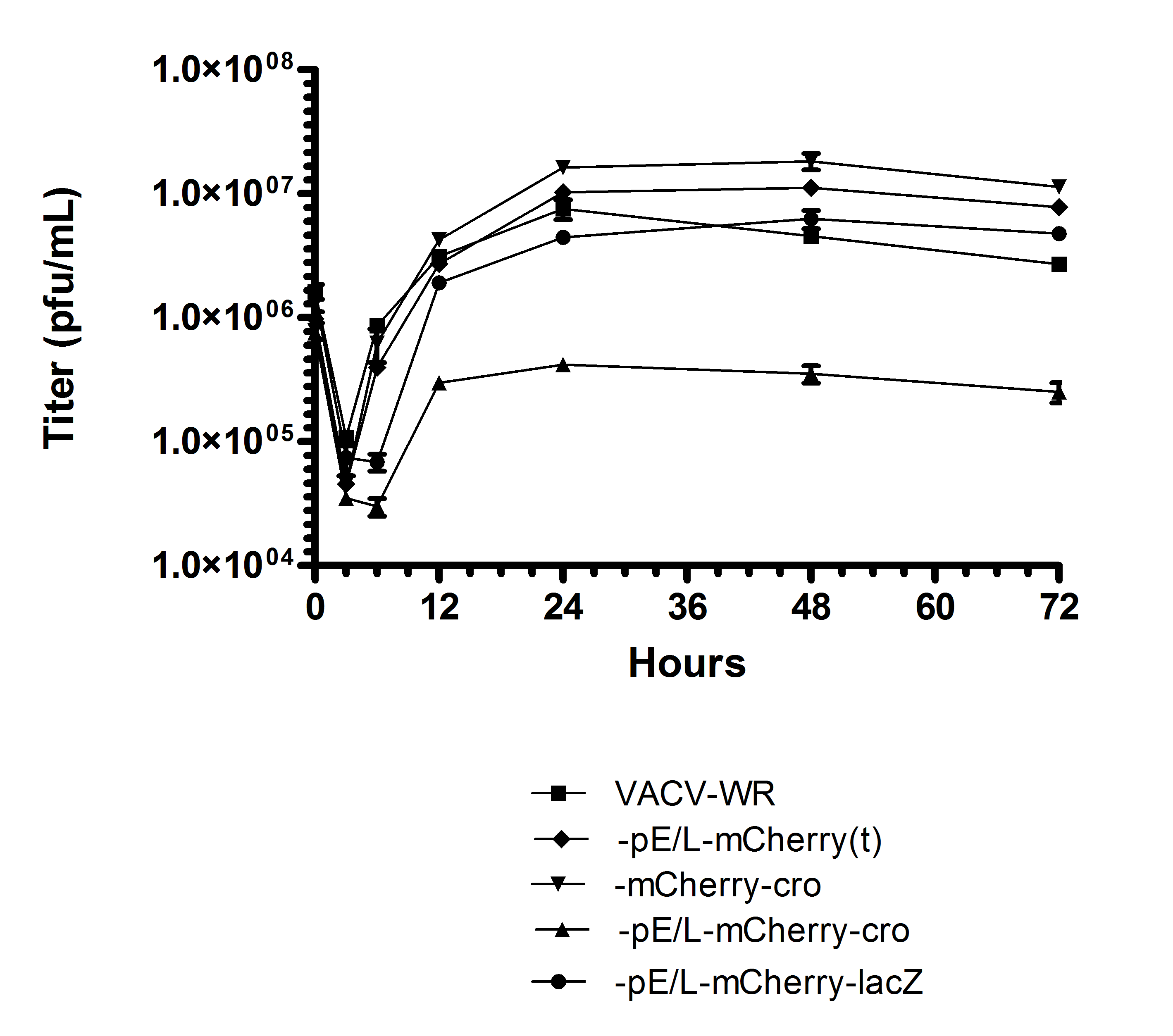

Supplement: S1 Fig — BSC-40 cells were infected with the indicated viruses at a MOI = 3. Viruses were harvested at the indicated time points and titered on BSC-40 cells. The mean ± S.E.M. from three independent experiments are shown. (TIF) [file ppat.1005824.s002.tif]

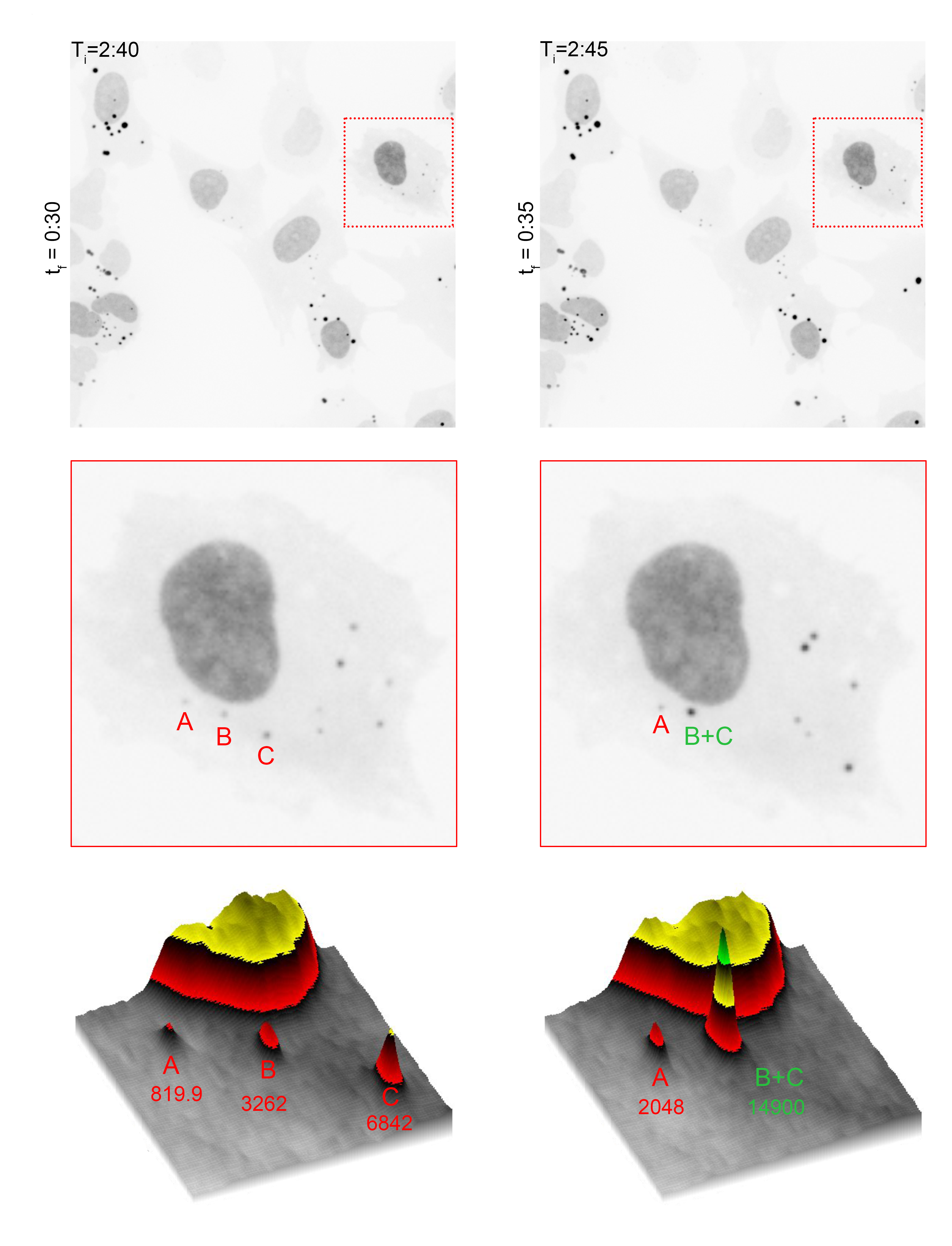

Supplement: S2 Fig — The two upper panels show two consecutive fluorescence images spanning 5 min. Fluorescence intensities from virus factories in the highlighted cell (red box) were quantified using FIJI software. The particles designated B and C seemed to fuse into a single larger and brighter particle (B + C). The two lower panels show the distribution of fluorescence in the above images. Numbers below labeled virosomes indicate the mean fluorescence intensities. (TIF) [file ppat.1005824.s003.tif]
