# Supplementary material for: Live-Cell Imaging of Vaccinia Virus Recombination
Source: PLoS Pathog. 2016 Aug 15;12(8):e1005824. doi: 10.1371/journal.ppat.1005824 (PMC4985154; doi:10.1371/journal.ppat.1005824)
Supplement: S1 References — (DOCX) [file ppat.1005824.s014.docx]

**References**

1. Lin YC, Evans DH. Vaccinia virus particles mix inefficiently, and in a way that would restrict viral recombination, in coinfected cells. Journal of virology. 2010;84(5):2432-43. Epub 2009/12/25. doi: 10.1128/JVI.01998-09. PubMed PMID: 20032178; PubMed Central PMCID: PMC2820930.

2. Irwin CR, Evans DH. Modulation of the myxoma virus plaque phenotype by vaccinia virus F11 protein. Journal of virology. 2012. Epub 2012/04/20. doi: 10.1128/JVI.06936-11. PubMed PMID: 22514354.

3. Wasilenko ST, Stewart TL, Meyers AF, Barry M. Vaccinia virus encodes a previously uncharacterized mitochondrial-associated inhibitor of apoptosis. Proceedings of the National Academy of Sciences of the United States of America. 2003;100(24):14345-50. PubMed PMID: 14610284.
